# The association between macro-level structural discrimination and alcohol outcomes: A systematic review

**DOI:** 10.1016/j.socscimed.2025.118596

**Published:** 2025-09-19

**Authors:** Sophie Bright, Charlotte Buckley, Daniel Holman, Madeleine Henney, Loni Philip Tabb, Robin Purshouse

**Affiliations:** 1 Sheffield Addictions Research Group, Sheffield Centre for Health and Related Research, School of Medicine and Population Health, University of Sheffield, UK; 2 Department of Psychology, Institute of Population Health, University of Liverpool, UK; 3 Department of Automatic Control and Systems Engineering, University of Sheffield, UK; 4 Department of Sociological Studies, University of Sheffield, UK; 5 Sheffield Centre for Health and Related Research, School of Medicine and Population Health, University of Sheffield, UK; 6 Dornsife School of Public Health, Drexel University, Philadelphia, United States

## 1 Background

### 1.1 Discrimination and alcohol outcomes

Alcohol consumption is a primary risk factor for both death and disability (Griswold et al., 2018) and has been causally linked to over 200 disease and injury conditions (Rehm et al., 2009). However, levels of alcohol consumption vary across populations (Bright et al., 2024). Further, individuals from disadvantaged backgrounds, such as those with lower socioeconomic status (SES) and minoritized racial and ethnic groups, bear a disproportionate burden of harm associated with alcohol, despite comparable or lower levels of consumption (Probst et al., 2021; Zapolski et al., 2014).

Discrimination likely contributes to these inequities. In their influential systematic review, Gilbert & Zemore (2016) examined the relationship between discrimination and alcohol-related outcomes and found a generally positive association: as experiences of discrimination increase, drinking, alcohol-related problems, and risk of disorders also tend to increase. However, although the authors aimed to include all types of discrimination, studies almost exclusively focused on interpersonal discrimination – i.e., perceived discrimination or unfair treatment in interactions with others —while structural discrimination remained notably underexplored.

A more recent systematic review and meta-analysis examined the association between racism-related experiences (e.g., self-reported historical, online, internalized and interpersonal discrimination) and different types of substance use (including alcohol) among ethnoracially minoritized 12- to 29-years-olds (Ebrahimi et al., 2024). The review found a significant positive association for both alcohol use and binge drinking. However, again, this review did not address structural discrimination.

Structural discrimination is increasingly being recognized as a fundamental driver of health inequities (Dean & Thorpe, 2022). It reflects the “totality of ways” in which societies foster discrimination through “mutually reinforcing systems of housing, education, employment, earnings, benefits, credit, media, health care, and criminal justice”, to “reinforce discriminatory beliefs, values, and distribution of resources” (Bailey et al., 2017). The small but growing body of reviews examining structural discrimination and health suggests a generally harmful impact. For example, Hailu et al. (2024) found that structural racism contributes to increased maternal morbidity and mortality, while Homan et al. (2021) found relatively consistent negative effects of structural sexism on women’s health. However, to our knowledge, no systematic reviews have specifically examined the relationship between structural discrimination—whether broadly defined or focused on a specific type—and alcohol-related outcomes. This is an important gap, as alcohol use represents a health behavior rather than a direct health outcome and may respond differently to structural forces. For example, some evidence suggests that alcohol use may be lower among women in high-sexism environments (Graham et al., 2020; McKetta et al., 2022, 2023).

Another key limitation of existing reviews, and/or the studies within them, is their narrow focus on a single type of discrimination. For example, in the review by Gilbert and Zemore, the vast majority of studies examined racism, with little attention paid to other forms, such as sexism or heterosexism. Further, very few studies explored how multiple forms of discrimination might intersect to shape alcohol-related outcomes. This siloed, single-axis approach is problematic, as it overlooks the reality that systems of oppression do not operate independently. As scholars of intersectionality have long emphasized, social positions such as race, gender, and class—and the systems of power associated with them, including racism, sexism, and classism—interact in complex ways that can compound disadvantage (Collins & Bilge, 2020; Crenshaw, 1989; Combahee River Collective, 1977). Notably, Bailey et al.’s (2017) definition of structural racism—which highlights the mutually reinforcing nature of discriminatory systems across domains—echoes Collins’ concept of the ‘matrix of domination,’ which describes how intersecting systems of oppression operate across structural, disciplinary, hegemonic, and interpersonal domains of power (Collins, 1991). Focusing on a single form of discrimination, or solely on individual or interpersonal levels, while neglecting discriminations interactive and systemic nature, therefore risks producing incomplete or misleading conclusions.

### 1.2 Structural discrimination: definitions and concepts

While structural discrimination can facilitate interpersonal discriminatory behavior, it is conceptually distinct and operates independently of individual intent (National Institute on Minority Health and Health Disparities, 2024). It is produced and sustained through institutional and governmental actions and policies—whether intentional or not. As Krieger (2020) explains, discrimination or ‘unjust isms’ (racism, sexism, heterosexism etc.) are supported at the structural level by the “explicit and non-explicit unjust ‘rules of the game’ (laws, policies, and rules), as well as area-based or institutional legacies and indicators of injustice.” For example, Jim Crow laws were designed to intentionally and explicitly undermine the rights of African Americans in areas such as housing, education and employment. Yet even ostensibly impartial practices can sustain or exacerbate structural inequities. For instance, many capitalist practices—such as basing mortgage provision on the availability of collateral—may appear to reflect sound business logic, yet still restrict opportunities for African American communities, who have historically been denied access to wealth-building opportunities (Corrigan et al., 2004; PINCUS, 1996).

It is also conceptually distinct from institutional discrimination, as it encompasses the interactions and cumulative effects across multiple institutions and systems (Bailey et al., 2017; Dean & Thorpe, 2022; Krieger, 2014). For example, the mass incarceration of Black people in the United States stems from overlapping and historically rooted discriminatory practices across the housing, economic, and criminal justice systems (Larrabee Sonderlund et al., 2022).

### 1.3 Structural discrimination: mechanisms

Structural discrimination operates differently to intra- and inter-personal discrimination, exerting a widespread impact that can affect entire communities or subgroups. Nancy Krieger’s ecosocial theory (Krieger, 2001, 2012, 2014) offers a useful framework for further understanding how structural discrimination can shape individual health outcomes.

According to ecosocial theory, we biologically incorporate the exposures we experience within our societal and ecological context. Discrimination can harm health through multiple interacting pathways of embodiment, operating across diverse spatio-temporal scales—from molecular to societal levels, and spanning history, intergenerationally, and across the life course. Pathways of embodiment include, for example, economic and social deprivation, social trauma, and targeted marketing of harmful commodities, and inadequate medical care.

There are numerous examples of how these pathways contribute to alcohol-related inequalities, particularly in relation to structural racism. For instance, historical redlining has led to ongoing racial residential segregation in the U.S., concentrating minoritized racial and ethnic groups in disadvantaged neighborhoods. These neighborhoods often have fewer health-promoting resources such as high-quality housing, educational services, green spaces, and healthcare (Swope et al., 2022). These areas are also more likely to have higher alcohol outlet density despite a lower consumer demand (Berke et al., 2010), and are disproportionately targeted by alcohol marketing (Grier & Kumanyika, 2010; Moore et al., 1996). Further, predominantly Black neighborhoods often experience heightened police surveillance – a form of social trauma that can contribute to harmful coping behaviors like drinking, particularly among Black youth (Jindal et al., 2022).

Conversely, structural discrimination may also suppress alcohol use through alternative mechanisms. For example, lower levels of drinking among African Americans may partly reflect their efforts to avoid racially biased and punitive societal repercussions (Zapolski et al., 2014). Additionally, racial segregation may reduce alcohol consumption in Black neighborhoods by reinforcing collective norms that discourage heavy drinking (Caetano & Clark, 1999).

### 1.4 Structural discrimination: measurement challenges and opportunities

While there are plausible mechanisms through which structural discrimination may influence alcohol-related outcomes, measuring structural discrimination and its impacts presents distinct challenges... First, in its fullest form, structural discrimination arises from interactions among multiple institutions and systems (Bailey et al., 2017; Dean & Thorpe, 2022; Krieger, 2014). In recent years there have been more attempts to quantify structural discrimination, with most employing macro-level measures, such as state- or neighborhood-level indicators of discrimination. However, though multiple domains – such as housing, socioeconomic status, criminal justice, and workplace practices – have been considered, they are often examined in isolation (Groos et al., 2018). For example, residential segregation has often been used as an indicator of structural racism (Dean & Thorpe, 2022). Such siloed approaches fail to capture overlapping and interacting effects of discriminatory societal structures. Therefore, to more accurately reflect this complexity, cross-domain index measures are recommended (Dean & Thorpe, 2022).

Second, obtaining accurate and comprehensive information on discriminatory laws and policies can be challenging. Such information is often inaccessible, and data on implementation and enforcement is frequently lacking. Further, some policies are not overtly discriminatory but are strategically designed to bypass anti-discrimination protections, further complicating efforts (Krieger, 2020).

Third, structural discrimination is embedded not only in formal policies, practices, and laws, but also in dominant cultural norms, values, and public discourse—underpinned by implicit hierarchies of superiority and inferiority. Population-level data on attitudes and beliefs that support structural -isms can be collected through surveys or analyses of online content (Krieger, 2020; Carlsson & Eriksson, 2017). However, the validity of such measures are limited by social desirability and implicit bias and aggregating individual-level attitudes may reflect interpersonal rather than structural discrimination.

Finally, disentangling structural discrimination from the mechanisms through which it affects health remains conceptually complex. For example, alcohol outlet density (AOD) can be understood as a direct exposure, reflecting structural racism through racially patterned zoning, licensing, and disinvestment. It may also operate as a mediator, explaining how residential segregation contributes to alcohol-related harm by increasing access and exposure to alcohol. Alternatively, AOD could function as an effect modifier, amplifying harm in segregated communities already burdened by chronic stress and limited protective resources.

Despite these challenges, addressing structural discrimination remains essential for reducing health inequities. Structural racism has been identified as a fundamental cause of health disparities, meaning inequalities in (alcohol-related) outcomes are unlikely to be resolved without tackling the underlying inequities in power, prestige, freedom, neighborhood conditions, and access to care linked to racism (Phelan & Link, 2015). Arguably, other forms of structural discrimination—such as sexism, classism, and heterosexism—also shape access to power, resources and opportunities, and may therefore influence health outcomes through similar pathways.

Studying structural discrimination also offers distinct advantages compared to interpersonal or intrapersonal forms. While the latter rely on individuals’ recognition of discriminatory events and society’s willingness to validate those experiences, structural indicators—such as the gender pay gap—are objective.. This objectivity may increase both the detectability of associations between structural discrimination and health outcomes and the likelihood of actionable policy responses. Similarly, studying the health impacts of anti-discrimination laws, policies, and practices (i.e., mitigation of structural discrimination) may support the development and implementation of evidence-based policy solutions.

### 1.5 Purpose of this review

As outlined above, most prior research has focused on interpersonal discrimination and typically examined a single axis—such as race—leaving important gaps in our understanding of how macro-level structural discrimination, across multiple forms, affects alcohol consumption and related health outcomes.

This review aims to address that gap by examining the association between structural discrimination of any type and alcohol-related outcomes. By maintaining a broad focus, it seeks to provide a comprehensive and policy-relevant synthesis of how structural forces shape alcohol-related harm. Specifically, we seek to answer, “What is the association between macro-level structural discrimination and alcohol consumption, and between macro-level structural discrimination and alcohol-related health outcomes?”

Critically, and as previously highlighted by Krieger (2014), this study does not intend to prove or disprove whether discrimination is harmful. It is well documented that discrimination is harmful to health (Selvarajah et al., 2022; Williams et al., 2019) and restricting the opportunities, power, and well-being of populations based on socially ascribed categories and/or arbitrary physical traits is inherently wrong, regardless of its impact on alcohol-related outcomes. Rather, the purpose of this study is to review existing literature on this relationship, better understand the role of structural discrimination in alcohol-related inequities and ultimately support action to address such inequities.

## 2 Methods

### 2.1 Information Sources

On March 21^st^, 2023, we searched four electronic bibliographic databases (PubMed, Embase, PycINFO and Sociological Abstracts) according to a pre-registered International Prospective Register of Systematic Reviews (PROSPERO) protocol (ID=CRD42023394379) and Preferred Reporting Items for Systematic Reviews and Meta-Analyses (PRISMA) methodology (Page et al., 2021). We limited our search to studies published since 1990, as a previous review of all types of discrimination found no relevant papers published prior to that (Gilbert & Zemore, 2016). Reference lists of included articles were manually screened to identify additional relevant studies. We also searched the Overton database for grey literature (e.g., policy documents, think-tank research) and reviewed the reference lists of relevant reports for additional studies meeting our inclusion criteria. Searches were re-run on 28 August 2024, prior to the final analysis, to capture more recent studies.

### 2.2 Population, Exposure and Outcome

Search terms were developed using the PEO (Population, Exposure, Outcome) framework (Moola et al., 2015). The population was human subjects, with no other restrictions.

The exposure of interest was macro-level structural discrimination. Drawing upon the definition of structural racism proposed by Bailey et al., (2017), we define this as the “totality of ways” in which societies foster discrimination through “mutually reinforcing systems of housing, education, employment, earnings, benefits, credit, media, health care, and criminal justice”, to “reinforce discriminatory beliefs, values, and distribution of resources”. This includes “explicit and non-explicit unjust ‘rules of the game’ (laws, policies, and rules), area-based or institutional legacies, and indicators of injustice” (Krieger, 2020). We acknowledge that structural discrimination differs from institutional discrimination in its emphasis on the interaction among multiple institutions and systems. However, these terms are often used interchangeably in the literature, and both refer to macro-level forms of discrimination (Braveman et al., 2022; Dean & Thorpe, 2022). Ultimately, our aim was to capture studies examining discrimination beyond the individual or interpersonal level.

Accordingly, we included studies regardless of the specific terminology used or whether the discrimination examined operated within a single system or across multiple systems.

The outcomes were i) any measure of alcohol consumption or drinking patterns (e.g., frequency, quantity, heavy episodic drinking) and ii) any alcohol-related health harm (e.g., alcohol-attributable mortality, liver cirrhosis). We did not include other types of harm (e.g., crime, loss of employment). See Table 1 for search terms.

### 2.3 Inclusion Criteria

We included studies that met all the following criteria:

- **Study Design**: Observational, quantitative epidemiological studies (e.g., cross-sectional, case-control, cohort, longitudinal).
- **Population**: Human subjects of any demographic group or geographic location.
- **Exposure**: Quantitative measures of macro-level structural discrimination, operationalized as any discriminatory laws, policies, and rules, area-based or institutional legacies, and indicators of injustice. We also included related macro-level indicators of equality (for example, policies designed to protect marginalized populations), considering this to equate to lower discrimination.
  We included any type of structural discrimination (including but not limited to racism, sexism, classism, ableism, xenophobia, or heterosexism), within any domain, (including but not limited to housing, education, employment, earnings, benefits, credit, media, health care, and criminal justice).
  We included studies regardless of whether authors used the term structural discrimination, provided they assessed discrimination at a macro level.
- **Outcome**:
  - (i) Alcohol consumption or drinking patterns (e.g., frequency, quantity, heavy episodic drinking), or
  - (ii) Alcohol-related health harms (e.g., liver cirrhosis, alcohol-attributable mortality).
- **Language**: English.
- **Timeframe**: 1^st^ January 1990 – 28^th^ August 2024.
- **Publication Type**: Peer-reviewed empirical literature.

### 2.4 Exclusion Criteria

Studies were excluded if they met any of the following criteria:

- **Discrimination Measure**: Focused solely on individual-level, interpersonal, or internalized discrimination (e.g., self-reported unfair treatment), or examined related but conceptually distinct constructs (e.g., social inclusion/exclusion, acculturation) without directly measuring structural or institutional discrimination. Studies using only individual or aggregate self-reported discrimination (e.g., unfair treatment in work, healthcare, or by police) were also excluded,
- **Study Design:** Qualitative studies, laboratory experiments, psychological simulations, or non-empirical works (e.g., editorials, commentaries, reviews).
- **Publication Type:** Conference abstracts, dissertations, or other non-peer-reviewed sources.
- **Language:** Not published in English.
- **Population:** Non-human research.

### 2.5 Article Screening, Data Extraction and Quality Assessment

Following removal of duplicates, the first author (SB) screened all titles and abstract, with a second reviewer (MH) independently reviewing 20% of the articles (overall agreement 92%). Both reviewers then screened full texts, reaching 84.8% agreement (Cohen’s K = 0.7). SB and CB extracted key study characteristics and outcomes, with MH cross-checking 20% (no disagreement). Where papers included sequential or hierarchical models, we extracted findings from the final, fully adjusted model. SB and CB assessed study quality using a modified version of the Newcastle Ottawa Scale for non-randomized studies (Wells et al., 2021). Any discrepancies in scoring or interpretation were resolved through discussion until consensus was reached, without the need for third-party adjudication. See supplementary materials for assessment criteria and further details on discrepancy resolution.

## 3 Results

### 3.1 Study characteristics

**Figure 1:**
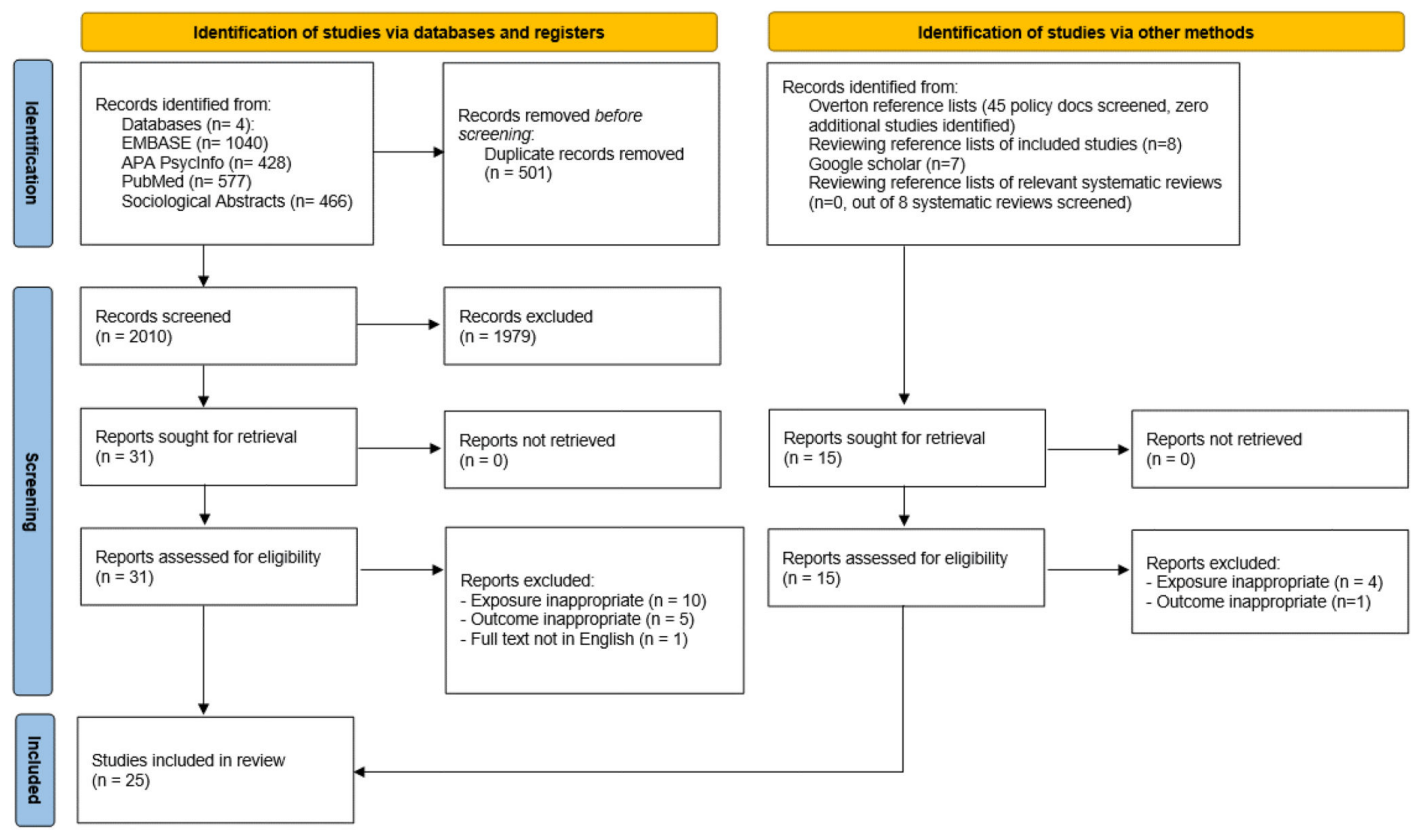
PRISMA flow diagram depicting the flow of information through the different phases of the systematic review

After removing 501 duplicates, we identified 2,010 articles from four databases. Following title and abstract screening, 1,979 articles were excluded, and 16 removed during full-text screening, leaving 15 studies. We found 10 additional studies through grey literature, Google Scholar, and reference list screening. These studies used different key terms to those included in our search, discussed further in the limitations section. Our final analytic sample included 25 studies (15 from database searches, 10 from other methods). Full details are provided in Figure 1.

Study characteristics are summarized in Table 2. The first study was published in 2006, with most published between 2020 and 2024. The most studied type of structural discrimination was racism (n=11), followed by sexism (n=7), and heterosexism (n=4). Three studies considered two or more types of discrimination simultaneously, referred to hereafter as ‘intersectional discrimination’. Most studies (n=21) were conducted in the US with the remainder making between-country comparisons (n=4). US studies mostly measured discrimination at the state level (n=11) or a sub-state level (e.g., district or census block, n=10). Most studies (n=17, 68%) focused on alcohol consumption outcomes, three (12%) on alcohol-related harms, and five (20%) on both. The most assessed outcomes were:

- **Heavy Episodic Drinking (HED):** Refers to consuming a large quantity of alcohol on a single occasion. While definitions vary, it is often defined as consuming five or more drinks on one occasion.
- **Drinking status:** Indicates whether the respondent consumed any alcohol within a specified period (e.g., past 30 days or past 12 months).
- **Drinking frequency:** The number of drinking occasions within a defined timeframe (e.g., number of days alcohol was consumed in the past month).
- **Alcohol Use Disorder (AUD):** Typically defined according to DSM-5 criteria as a pattern of problematic alcohol use over a 12-month period, characterized by symptoms of impaired control, social impairment, risky use, and physiological dependence, resulting in significant impairment or distress.
- **Alcohol volume:** Estimated using quantity–frequency approaches, in which measures of typical drinking quantity are combined with drinking frequency over a specified period to generate an overall estimate of alcohol consumption.

Less frequently assessed outcomes included ‘heavy drinking’, ‘high-intensity drinking’, ‘volume from heavy drinking’, ‘risky drinking’, ‘alcohol abuse’, and ‘alcohol abuse disorder’. While some of these terms appear similar, their operational definitions varied slightly across studies; each is therefore defined within the results where relevant.

Based on the modified Newcastle-Ottawa Quality Assessment, eleven studies were classified as ‘good’ quality, thirteen ‘fair,’ and one ‘poor’. Due to study heterogeneity, meta-analysis was not appropriate.

#### 3.1.1 Analytical approaches

The most common analytical approach, used in 13 studies, was multilevel regression – used to account for the nested structure of the data, typically including random intercepts to model clustering of individuals within higher-level units such as schools, states, or countries. These approaches were particularly common in studies using longitudinal data or data drawn from multi-site sampling designs. Single-level regression models were used in 8 studies, most often when the intraclass correlation coefficients were found to be negligible or when the data did not warrant hierarchical modelling. Structural equation modelling was used in four studies. These models were used to estimate direct and indirect effects, account for latent constructs or explore interactions. One ecological study used a correlation-based approach, examining associations at the country level.

Almost all studies adjusted for a broad set of individual-level sociodemographic variables. The most common of these included age, gender or sex and race and/or ethnicity. Many studies also controlled for socioeconomic indicators such as educational attainment, household income, employment status, and marital or partner status. In studies involving adolescents or young adults, studies often included family variables such as parental education, parental marital status, family structure and household composition. Studies focusing on LGBTQ+ populations included measures of sexual and gender identity and sometimes relationship status, housing instability, or military service, depending on the population studied. A total of 18 studies controlled for at least one macro-level or contextual variable. These varied depending on the level of analysis and research question. For example, at the state or country level, macro-level covariates included measures such as gross domestic product (GDP) or gross national income (GNI) and the GINI coefficient, a measure of income inequality, while at the neighborhood or census block group level, they included factors such as neighborhood unemployment, racial composition and population density.

### 3.2 Results by type of structural discrimination

Study findings are summarized in Tables 3-6, categorized by racism, sexism, heterosexism, and intersectional discrimination, respectively. A positive association between discrimination and a given alcohol outcome indicates that higher levels of discrimination are associated with increases in that outcome. For consistency we report findings as significant at the 0.05 level unless otherwise stated.

#### 3.2.1 Structural racism

Eleven papers examined structural racism and alcohol outcomes (Chiang et al., 2024; Dudovitz et al., 2021; Egede et al., 2024; Karriker-Jaffe et al., 2013; Kim et al., 2022; LaVeist et al., 2008; Schwartz et al., 2022, 2023; Seffrin, 2012; Wang et al., 2022; Woodard et al., 2024), making it the most frequently studied type of discrimination in this review. All were US based, with nine measuring discrimination below the state-level (e.g., district, neighborhood, school) and two measuring state-level discrimination. Six studies were longitudinal, and the remaining five were cross-sectional. Most studies used single measures of structural racism (primarily racial segregation), with one using a composite measure. Nine studies focused on alcohol consumption, one on peers’ consumption, one on alcohol-related harm, and one on both. Seven reported outcomes for one or more racially minoritized groups plus White people, while four focused solely on Black individuals.

##### 3.2.1.1 Racial segregation and drinking status

Five studies considered the association between racial segregation and drinking status, with inconsistent results. Among Black Americans, two studies found a potentially positive association, one found no association, and two suggested a negative association.

Two longitudinal studies, both using data from the Panel Survey of Income Dynamics (PSID), considered the association between district-level school racial segregation and the probability of ever drinking among Black Americans. Both used the Black-White dissimilarity index, indicating the proportion of Black/White students who would need to switch schools to achieve an even racial distribution, and also applied Instrumental Variable (IV) models to address potential confounding, using time since court-ordered desegregation as the instrument.. The first study focused on Black children aged 5-17 (Wang et al., 2022). No associations were found with the dissimilarity index, but in IV models increased school segregation was associated with increased likelihood of ever drinking. The second study focused on Black adults aged 18-44 (Kim et al., 2022). Similarly, no association was found for the dissimilarity index whereas IV analysis suggested a positive association, though this was not robust after adjusting for multiple hypothesis testing.

A third study, also using PSID data, explored associations between childhood residential racial segregation trajectories and young adult health outcomes for Black Americans, and found no significant associations with ever drinking (Schwartz et al., 2022).

The remaining two studies suggested a negative association for Black Americans. Chiang et al., (2024) found that higher district-level school segregation was associated with lower risk of ever drinking alcohol, but only among Black adolescents attending majority non-White schools. Later in adulthood (mean age 28), associations with the same outcome were non-significant. LaVeist et al. (2008) found no significant difference in the odds of being a current drinker between White people and African Americans in a racially integrated community. However, in a more segregated community (a matched U.S. sample), African Americans had significantly lower odds of being current drinkers compared to White individuals.

##### 3.2.1.2 Racial segregation and Heavy Episodic Drinking (HED)

Six studies examined the association between racial segregation and HED, with mixed findings: two reported a negative association, one a positive association, and three found no association.

Chiang et al., (2024) observed that higher district-level school segregation was associated with lower frequency of drinking to the point of drunkenness for Black respondents attending majority non-White schools. Similarly, Egede et al. (2024), using structural equation modelling, found that historic redlining was directly associated with a reduced prevalence of HED at the census tract level.

In contrast, Kim et al. (2022), reported a positive association between school racial segregation and the probability of HED among Black adults. This was observed for both exposure measures (the Black-White dissimilarity index and their IV), and the association remained robust after adjustments for multiple hypothesis testing.

Three studies found no association between segregation and HED among Black Americans. Woodard et al. (2024) found no significant association between state-level residential segregation and monthly HED frequency. Wang et al. (2022) found no significant association between school segregation and their binary monthly HED variable. Similarly, Schwartz et al. (2022) found no association between childhood residential racial segregation trajectories and annual HED frequency.

##### 3.2.1.3 Racial segregation and drinking frequency

Four studies examined the association between racial segregation and drinking frequency, again with mixed findings. Two studies suggested a positive association among Black Americans, one found the association to depend upon the racial composition of the segregated community, and one reported no association.

Schwartz et al. (2022) observed that Black young adults who lived in highly segregated neighborhoods throughout their childhood had higher odds of drinking more than once a week compared to those who always lived in lower-segregation neighborhoods. Wang et al. (2022) found no associations with drinking at least monthly for their Black-White dissimilarity index exposure, but found a positive association using their IV. When stratified by sex, this positive association held for Black girls but not Black boys.

Seffrin (2012) found that the impact of segregation on adolescent drinking frequency varied by community racial composition.. They examined changes in adolescent drinking over time at three levels of segregation (0%, 50% and 75% Black), controlling for individual race and other risk and protective factors. They found drinking frequency increased least in highly segregated Black communities (75% Black), increased moderately in mixed neighborhoods, and. increased the most in highly segregated White communities (0% Black).

Woodard et al. (2024) found no association between state-level residential segregation (averaging scores from the index of dissimilarity and the isolation index) and past-month drinking frequency among Black adult drinkers.

##### 3.2.1.4 Racial segregation and alcohol abuse

Dudovitz et al. (2021) assessed the association between school-based segregation and ‘alcohol abuse’, defined as two or more episodes of alcohol-related life problems. They found that, controlling for neighborhood racial composition, attending a school with a higher percentage of White students was associated with increased alcohol abuse among White students, but not among Black or Latinx students. Additionally, greater racial and ethnic cohorting within schools was associated with lower alcohol abuse among Black and Latinx students, but higher alcohol abuse among White students.

##### 3.2.1.5 Racial segregation and peer alcohol consumption

Schwartz et al. (2023) assessed peer alcohol consumption. While overall associations were non-significant, subgroup analyses revealed important differences. For Black students in predominantly White schools, higher segregation was associated with a higher peer alcohol use (ever having ever drank and frequency of drinking (positive association). In contrast, for non-Black students of color in predominantly non-White schools, higher segregation was associated with less peer drunkenness (negative association). No significant associations were found for White students.

##### 3.2.1.6 Other structural racism exposures

Two studies considered alternative (non-segregation) structural racism outcomes, finding several positive associations for minoritized groups. Karriker-Jaffe et al., (2013) found that higher income inequality measured using between-race (Black-White and Hispanic-White) poverty ratios was associated with increased volume from both light and heavy drinking among Black, Hispanic and White people. They also found moderated associations in relation to negative alcohol-related consequences and alcohol dependence, such that higher income-inequality was associated with greater consequences and dependence for Black and Hispanic people than for White people.

Woodard et al. (2024) explored associations between monthly drinking and HED frequencies and the Black-White incarceration rate gap, the educational attainment gap, the economic disparity index, and the employment disparity index, plus a composite measure combining all indicators. They found no association between their composite measure (or for most of its comprising individual indicators) and either outcome. However, Black Americans in states with the highest incarceration rate gaps had significantly higher HED frequencies (approximately nine more HED events per year) than those in states with the lowest.

##### 3.2.1.7 Summary

Most studies of structural racism used single indicator exposures, primarily measures of racial segregation. However, findings related to segregation were highly variable, even when focused on the same alcohol outcome and racial subgroup. In contrast, the two studies considering other forms of racism found some positive associations for specific exposures. Higher race-based poverty ratios were associated with increased volume, negative alcohol related consequences, and dependence – particularly for Black and Hispanic populations. Similarly, racial inequalities in incarceration were associated with increased HED among Black Americans. While few studies considered variation by gender, the limited evidence suggests there may be differential effects.

#### 3.2.2 Structural sexism

Seven studies examined structural sexism and alcohol outcomes (Bond et al., 2010; Graham et al., 2020; Kuntsche et al., 2011; McKetta et al., 2022, 2023; Rahav et al., 2006; Roberts, 2012), making it the second most frequently studied type of discrimination.

Four studies made comparisons across countries, while three compared US state-level measures. Unlike the studies on structural racism, only two were longitudinal, and all but one used composite exposure measures, though their comprising indicators for these measures varied. All studies assessed alcohol consumption, often reporting on multiple outcomes in a single study. One study also evaluated alcohol-related health harms. Five considered the impact for both men and women, while two focused on women only.

Gender equality refers to “the entitlement of all genders to enjoy equal rights, opportunities, and treatment” (Milner et al., 2021). Its absence—that is, gender inequality— can therefore also be considered an indicator of gender-based discrimination or structural sexism. As a result, scholars have framed similar indicators as measures of either gender equality or gender-based discrimination. For example, McKetta et al. (2023) use the male-to-female ratio in the labor force as an indicator of structural sexism, while Roberts (2012) use a comparable indicator—the ratio of women to men participating in the labor force—as a measure of gender equality. We therefore refer to both ‘structural sexism’ and ‘gender equality’ when presenting results, to reflect the terminology used in the related study. When reporting associations, we refer to associations with sexism, interpreting lower levels of gender equality as indicative of higher levels of structural sexism.

All studies relied on binary gender classifications (male/female or men/women), with no reference to the inclusion of transgender or non-binary individuals. The applicability of these findings to gender-diverse populations is therefore unclear.

##### 3.2.2.1 Sexism and drinking status

Two studies examined the relationship between gender equality and drinking status, both suggesting that greater gender equality (lower structural sexism) is associated with higher odds of being a drinker, for both men and women (i.e., a negative association).

Graham et al. (2020) analyzed survey data from the multi-country ‘Gender, Alcohol, and Culture: An International Study’ (GENACIS) project, using multilevel modelling to account for individuals being clustered within countries. They examined the association between country-level gender inequality and drinking status. Greater gender equality was associated with higher odds of being a drinker for both women and men, particularly women (OR = 2.1 for women; 1.6 for men per one-point increase in equality). No significant interaction was found between gender equality and living with children…

Roberts (2012) also used multilevel modelling to examine associations between five separate state-level gender equality indicators (e.g. equality in reproductive rights, each a composite measure) and drinking outcomes in the U.S.. In models adjusting for individual-level covariates, all gender equality indicators were positively associated with the odds of being a drinker, for both men and women (ORs up to 1.26 and 1.30 respectively).

Associations were strongest for women’s socioeconomic status but all became non-significant after adjusting for additional state-level characteristics, such as income inequality and religious composition.

##### 3.2.2.2 Sexism and Heavy Episodic Drinking

Three studies examined the relationship between structural sexism and HED. Two studies, using aggregate measures of structural sexism and reported on mediators and moderators of this relationship. The other found a positive association for specific indicators.

McKetta et al. (2022, 2023) analyzed longitudinal data on women from the Monitoring the Future (MTF) Panel study, using three-level random-intercept models, with observations nested within individuals, nested within states. They measured state-level sexism using a time-varying composite gender inequality measure, and considered the odds of HED over last 2 weeks. McKetta et al. (2022) found that higher levels of structural sexism were associated with lower odds of HED among women (fully adjusted OR: 0.917), with restrictive alcohol norms partially mediating this relationship. Secondary analyses of men found the same, but weaker, negative association (fully adjusted OR: 0.976). McKetta et al. (2023) also examined how structural sexism moderated the relationship between occupational characteristics and HED among women. Overall, employed women and those in high-status occupations had higher odds of HED compared to non-working women. However, as sexism decreased, the odds of HED increased faster among employed women, widening this disparity between the employed and unemployed.

Roberts (2012) also assessed associations with HED, but as a continuous variable (number of HED days). In fully adjusted models, they found that as gender equality relating to reproductive rights and violence policy decreased (i.e., sexism increased), HED increased among women. No significant associations were found for men.

##### 3.2.2.3 Sexism and drinking frequency

The same three studies that analyzed HED also considered monthly drinking frequency.

McKetta et al. (2022, 2023) found a negative association; higher structural sexism was associated with lower drinking frequency (RR: 0.974). This relationship was partly explained by restrictive alcohol norms and college completion (McKetta et al., 2022). In lower sexism states, frequency increased more rapidly among employed women and those in high-status occupations compared to unemployed women, similarly to their findings for HED (McKetta et al., 2022).

In fully adjusted models, Roberts (2012) found a significantly positive association between structural sexism and drinking frequency for both men and women, although only for their socioeconomic equality indicator. The effect varied by education level, with women in high-equality states drinking more overall, and those with college education showing the strongest association.

##### 3.2.2.4 Sexism and alcohol volume

Two studies investigated the relationship between structural sexism and average volume of alcohol consumption. Neither found significant associations for women, however there was some evidence of a potentially positive association for men, particularly those living with children.

Roberts (2012) analyzed the relationship between state-level gender inequality and average daily consumption among drinkers. In fully adjusted models, no significant association between was found for women. However, greater socioeconomic gender equality (lower structural sexism) was associated with lower alcohol consumption volume for men.

Graham et al. (2020) examined whether country-level gender inequality influenced the relationship between living with children and alcohol volume. Among women, living with children was associated with lower alcohol consumption, regardless of gender equality. For men, the association varied: in lower sexism countries, living with children was associated with lower alcohol consumption, while in higher sexism countries, men with children drank slightly more, though not significantly.

##### 3.2.2.5 Sexism and drinking quantity

One study assessed drinking quantity, defined as the usual quantity consumed on a drinking day, measured in grams of pure alcohol (Kuntsche et al., 2011). Using data from the GENACIS project, the study applied increasingly complex multi-level models to investigate how individual factors (engagement in paid labor and relationship status) along with country-level income equity (gender–income ratio), influenced alcohol consumption among mothers who drink.

Among partnered non-working mothers (‘housewives’) – there was no significant association between structural sexism and drinking quantity. Among partnered mothers, working was associated with higher quantities of alcohol consumption than not working. However, the detrimental effect of employment on the usual quantity of partnered mothers was moderated by country-level income equity. I. n less equitable (more sexist) countries, partnered working mothers drank more than housewives, however in countries with lower structural sexism housewives drank more.

##### 3.2.2.6 Sexism and risky drinking

One study examined ‘risky drinking’, defined as. at least one HED occasion in the past 30 days, plus a 30-day alcohol volume exceeding 30 grams for women and 60 grams for men (Roberts, 2012). Structural sexism related to violence policy showed a positive association with risky drinking among women, while structural sexism concerning reproductive rights was positively associated with risky drinking among men. Among women, effects varied by education: those with college education had higher odds of risky drinking in high equality states, while those without had higher odds in less equal states.

##### 3.2.2.7 Sexism and alcohol-related health harms

Rahav et al. (2006) used GENACIS data to examine the relationship between structural sexism and alcohol-related health harms, considering two exposures: the United Nations’ Gender Empowerment Measure (GEM), and a self-developed Gender Equality Score (GES), which combines the GEM with additional measures. They found that higher GEM and GES (lower sexism) was associated with lower liver disease and cirrhosis death rates, and total alcohol death rates, for both men and women. GES was also positively associated with alcohol dependency death rates for women but not for men.

##### 3.2.2.8 Sexism and gender differences in drinking outcomes

Two studies using data from the GENACIS project considered gender differences in drinking outcomes, with varying findings.

Rahav et al. (2006) found that improvements in GEM and/or GES were associated with reduced gender differences in several alcohol-related outcomes, including the percentage of current drinkers, liver disease and cirrhosis death rates, alcohol dependency death rates, and motor vehicle crash death rates. Similar trends were observed for the percentage of weekly drinkers, heavy drinkers, and binge drinkers, though these findings were non-significant. Importantly, it is not clear from this study whether the reduction in gender differences was due to increased outcomes amongst women, reduced outcomes amongst men, or a combination of both.

Bond et al. (2010) examined the relationship between six gender equality exposures (each modelled separately) and gender differences in public and private drinking frequency.

Greater equality in empowerment, economic participation, reproductive autonomy and educational attainment, and the context of violence against women, predicted *smaller* gender differences in *public* drinking, though only equality in economic participation remained significant after accounting for Gross Domestic Product (GDP). Conversely, greater educational equality was associated with *larger* gender differences in *private* drinking, though this was again non-significant after GDP adjustment.

##### 3.2.2.9 Summary

All but one study of structural sexism used composite exposure measures. Most found some significant associations, though the direction of associations varied depending on the specific exposure and outcome examined. While there were too few comparable studies to make definitive conclusions, the. available evidence indicates that as gender equality increases (structural sexism decreases), the likelihood of a woman being a ‘drinker’ seems to increase, while risky drinking and alcohol-related mortality may decrease. Findings were mixed in relation to HED and drinking frequency amongst women, with a broad composite of gender inequality suggesting negative associations, but more narrow composites relating to specific aspects of sexism indicating positive associations. Findings for volume and quantity were largely non-significant.

Studies considering interaction effects suggest that associations vary by other individual characteristics such as women’s employment status and level of education, and men’s parental status. The two studies examining gender differences in outcomes suggest that greater gender equality is associated with smaller gender differences in the frequency of drinking in public setting and alcohol-related mortality.

#### 3.2.3 Structural heterosexism

Four studies investigated structural heterosexism (Drabble et al., 2022; Everett et al., 2016; Hatzenbuehler et al., 2009, 2010). All were US-based and measured structural discrimination at the state-level. All measured discrimination as exposure to different policy environments, either comparing states with fewer anti-discriminatory policies to those with more or comparing outcomes before and after a specific policy was introduced. One study was longitudinal, and the remainder cross-sectional. Two considered both consumption and harm outcomes, and two considered harms alone. Three studies considered the impact of heterosexism for sexual minority and heterosexual individuals (stratified by gender in one instance), and one assessed outcomes for a specific minoritized group only (lesbian women).

##### 3.2.3.1 Heterosexism and Alcohol Use Disorder

One study found a positive association between structural heterosexism and AUD for sexual minority groups (Hatzenbuehler et al., 2010), while two found no significant associations (Drabble et al., 2022; Hatzenbuehler et al., 2009).

In their first, cross-sectional study, Hatzenbuehler et al. (2009), investigated the modifying effect of state-level policies protective against sexual orientation discrimination, on the association between lesbian, gay, or bisexual (LGB) status and the prevalence of AUD. They found that living in states without policies extending protections (higher discrimination), compared with living in states with these policies (lower discrimination), predicted a stronger association between LGB status and AUD in the past 12 months. However, this association was not-significant.

In their second, longitudinal study, Hatzenbuehler et al. (2010), compared the prevalence of AUDs over time in states with constitutional amendments banning gay marriage (16 states) to those without such amendments (34 states). They found a significant increase in AUD prevalence among LGB respondents living in states that introduced marriage bans, with a 41.9% increase between the two survey waves, the second of which coincided with the passing of the amendments. In contrast, there was no similar increase among LGB respondents in states without the amendments. Additionally, the study found no comparable increase in AUD prevalence among heterosexuals living in states with constitutional amendments.

Drabble et al. (2022) used data from the National Alcohol Survey (NAS) and applied gender-stratified logistic regression models to examine how the heterosexism policy environment differentially affects AUD by sexual identity. They created a time-varying index of ten policies relevant to sexual minorities, defining states with four or more policies as having ‘comprehensive protections’. Their analysis found no association between comprehensive policy protections and AUD for heterosexual or sexual minority men and women.

##### 3.2.3.2 Heterosexism and High Intensity Drinking

Drabble et al. (2022) also considered associations with a binary High Intensity Drinking (HID) variable, with HID defined as eight or more drinks in a day at least once in the past year (HID vs no HID). They found that comprehensive policy protections (lower heterosexism) was associated with a lower probability of HID among sexual minority men. Similar associations were found for sexual minority women and heterosexual men, but these were non-significant.

##### 3.2.3.3 Heterosexism and Heavy Episodic Drinking, intoxication, consequences, and dependence

Everett et al. (2016) explored associations between civil union legislation and four other alcohol outcomes: HED, frequency of subjective intoxication, alcohol-related consequences, and symptoms of potential alcohol dependence. They analyzed alcohol outcomes in cross-sectional samples of Black, Latina, and White lesbian women at three time points related to the legalization of civil unions (before the bill was passed, between the passage and enactment, and after the bill was enacted) and included interactions for level of education and for race/ethnicity.

They found that alcohol-related consequences decreased after the bill was signed and after it was enacted, with no significant subgroup effects. Among those with a high school education or less HED also decreased after the bill was signed, however, more broadly HED increased after the bill was enacted.. No association was found for intoxication frequency overall, but Black and Latina women reported lower intoxication frequency compared to White women after the bill was signed. No association was found for symptoms of alcohol dependence overall., but White women reported increased symptoms after the bill was passed.

##### 3.2.3.4 Summary

In summary, there is some evidence to suggest there may be a positive association between structural heterosexism and AUD for sexual minority groups, and between heterosexism and HID for sexual minority men. There is insufficient evidence on other outcomes at present, but associations appear to vary by gender, race, and level of education.

#### 3.2.4 Intersectional discrimination

Three US-based studies considered the impact of multiple forms of structural discrimination simultaneously (Blosnich et al., 2016; English, 2021; English et al., 2022), two considering consumption (heavy drinking) and one harm (AUD).

##### 3.2.4.1 Heavy drinking

English (2021) and English et al. (2022) conducted two cross-sectional studies on heavy drinking among Black and White sexual-minority men, using structural racism (State Racism Index), and hetero/cissexism (State Equality Index) as exposures. The first considered men aged 16+, while the second restricted the sample to those aged 16-25. while. Both studies found that for Black participants, structural racism was positively associated with heavy drinking, with stronger effects in states with more anti-LGBTQ policies.. In the study with the younger sample, anti-LGBTQ policies were also positively associated with heavy drinking for Black participants. No significant associations were found for White participants in either study.

##### 3.2.4.2 Alcohol Abuse Disorder (AAD)

Blosnich et al. (2016) examined the association between indicators of community- and state-level Lesbian, Gay, Bisexual, and Transgender (LGBT) equality and diagnosed AAD (based on ICD-9 diagnostic criteria), among a purposive sample of transgender veterans (n=1,640). The exposures were i) binary indicators of whether transgender status or gender identity were covered by state employment non-discrimination and hate crime laws, and ii) the Municipal Equality Index (a city level composite with higher scores indicating greater equality for LGBT individuals).. They found no significant associations for either exposure.

##### 3.2.4.3 Summary

In summary, there is scant evidence considering multiple forms of discrimination simultaneously. However, some evidence suggests that different types of structural discrimination may interact in relation to heavy drinking, with compounding negative effects.

## 4 Discussion

### 4.1 Summary of findings

This systematic review sought to answer, “*What is the association between macro-level structural discrimination and alcohol consumption, and between macro-level structural discrimination and alcohol-related health outcomes*?” In doing so, we provide a comprehensive overview of current evidence on how several types of structural discrimination influence alcohol outcomes.

We found that the relationship between structural discrimination and alcohol-related outcomes varies considerably depending upon the type of discrimination examined, how it is measured, the demographics of the exposed population, and the specific alcohol outcome assessed.

Racism was the most frequently studied form of discrimination. Most studies focused on racial segregation using single-indicator measures, and findings varied widely—even when assessing the same outcomes within the same racial subgroups. However, emerging evidence suggests that other forms of structural racism, such as race-based poverty ratios and racial gaps in incarceration, may be associated with increased alcohol use and harm among Black and Hispanic populations.

Studies of structural sexism most often used composite or ‘index’ measures (e.g. state-level gender inequality indices). While research in this field remains highly heterogeneous, the available evidence suggests that as gender equality increases, women are more likely to drink. Conversely, structural sexism may be linked to higher rates of risky drinking and alcohol-related mortality. Findings on HED and drinking frequency were mixed: broader composite measures of sexism suggested negative associations with HED, while more narrow composites capturing sexism within specific domains (e.g., reproductive rights and violence policy) showed positive associations. Results for volume and quantity consumed were largely non-significant.

Studies of structural heterosexism all considered different policy environments as exposures, potentially reflecting the recent and explicit legal and policy changes regarding LGBTQ+ rights. The limited available evidence suggests a positive association between structural heterosexism and AUDs among sexually minoritized groups overall, and with HID for sexual minority men.

Only a few studies examined multiple forms of structural discrimination simultaneously, but early evidence suggests that one form of structural discrimination may amplify the effects of another.

Substantial heterogeneity in terms of study populations, contexts, and analytic strategies prevented further meaningful grouping of findings, for example by specific exposure measures, population level, or study designs. Moreover, even if such groupings were possible, the findings were too mixed to support the identification of consistent trends.

### 4.2 Potential mechanisms and explanations

#### 4.2.1 Racism

Conflicting findings in relation to the impact of racial segregation may reflect differences in sample characteristics (e.g., age groups), the racial composition of the segregated community (i.e., whether primarily White or Black), methodological approaches (e.g., instrumental variables vs. direct measures of segregation), or unexamined confounding factors. However, there are also plausible mechanisms through which structural racism could increase or decrease alcohol consumption and related harm.

For one, predominantly Black communities experience disproportionate levels of police surveillance. While this heightened surveillance may discourage alcohol consumption due to fear of police encounters, such encounters themselves have also been associated with increased substance use among Black youth (Jindal et al., 2022). Further, neighborhoods with a predominance of racially minoritized populations often lack essential resources necessary for health promotion (such as access to green spaces, high-quality education and healthcare) and have a higher density of alcohol outlets despite a lower demand for alcohol (Berke et al., 2010), both of which may increase alcohol consumption and related harms.

Conversely, some studies suggested that racial segregation reduces alcohol consumption among Black individuals in predominantly Black environments, while increasing it among White individuals in predominantly White environments. This may reflect how segregation reinforces culturally embedded drinking norms tied to racial identity. For example, Black and Hispanic Americans tend to consistently report more conservative or “drier” norms than White Americans (Caetano & Clark, 1999; Zapolski et al., 2014). In addition, protective resources in Black communities –such as proscriptive religiosity and family social support– may encourage abstinence and discourage heavy drinking (Mulia et al., 2018). In contrast, predominantly White environments may lack comparable protective social controls and may reinforce heavier drinking as normative. A lack of intergroup mixing due to segregation may therefore limit exposure to alternative perspectives on alcohol use, as well as access to societal structures that support abstention or moderation.

The findings for other forms of structural racism – racial gaps in incarceration and poverty rates – are consistent with the literature on interpersonal racism. That is, increased discrimination is associated with increased alcohol consumption and related consequences among minoritized populations (Ebrahimi et al., 2024; Gilbert & Zemore, 2016). Unlike segregation, structural discrimination that more overtly disadvantage minoritized groups, such as inequities in incarceration, may be particularly likely to drive alcohol use as coping response to systemic oppression.

Notably, only one study considered how associations may vary at the intersection of race and gender, highlighting an important gap in the literature.

#### 4.2.2 Sexism

Greater gender equality (lower. structural sexism) appears to be associated with higher likelihood of being a drinker., particularly for women. Several pathways may explain this relationship. Increased gender equality can lead to a convergence in gender norms and a reduction in the social stigma surrounding women’s drinking. Additionally, as women attain similar educational and professional status as men, they may gain greater social and economic independence, enabling more autonomous choices about consumption and leading to drinking patterns that more closely resemble those of men (McKetta et al., 2022, 2023).

However, though gender equality may normalize and facilitate moderate alcohol use among women, sexism may contribute to risky drinking. One possible explanation for this is that, in sexist societies, women may drink excessively in response to reduced autonomy, limited financial and social freedoms, and less fulfilment from diverse roles (Roberts, 2012).

Further, some evidence suggests structural sexism may be associated with increased alcohol-related mortality for both men and women. Structural sexism may limit women’s ability to seek help for alcohol-related problems, with evidence showing that higher state-level structural sexism is associated with reduced healthcare access and greater affordability barriers for women (Rapp et al., 2022). Further, from a broader gendered power and resource allocation perspective, sexist environments disempower women, setting in motion social, political, and economic dynamics that limit access to health-promoting resources for all (Dore et al., 2024). Similarly, in relation to HED, positive associations were observed specifically in the context of sexism in reproductive rights and violence policy. This may may reflect the use of HED as a coping response to stressful or traumatic experiences, such as being denied abortion access (Post et al., 2024), or intimate partner violence (Plichta, 2004; Ullman et al., 2005).

These findings align with broader research showing that regions with higher levels of gender equality tend to have better health outcomes (King et al., 2020; Milner et al., 2021). Further research is needed to clarify these relationships, but existing evidence suggests that concerns about alcohol-related harms among women should not be used to justify opposition to policies that advance gender equality (Roberts, 2012).

Importantly, studies considering interaction effects suggest that the association between sexism and alcohol-related outcomes varies by individual characteristics, including employment status, education level, marital status, and parental status (Graham et al., 2020; Kuntsche et al., 2011; Roberts, 2012). For example, Roberts (2012) found that women with higher education tend to drink more in high equality states than in low equality states, whereas women with lower education drank more in low equality states. These patterns may stem from varying social and economic pressures. Women with lower education in low equality states often face heightened financial and social constraints, which may drive alcohol consumption as a coping mechanism. Even in more egalitarian environments, these women may still encounter systemic barriers that limit their social participation and, in turn, temper their drinking behavior. Generally, it is recognized that while drinking has become more socially accepted for some groups over time (such as White women in privileged classes), women with lower SES—especially racially and ethnically minoritized women— continue to face greater surveillance, stigmatization, and penalization for alcohol use (Schmidt, 2014). In contrast, women with higher socioeconomic status in more gender-equal US states may experience fewer social constraints and greater financial freedom, increasing both their opportunity to drink, and its social acceptability.

Social roles also appear to influence the relationship between gender equality and alcohol use. Individuals occupying multiple roles —such as parent, partner, and employee— have role-related responsibilities that may reduce their capability, opportunity or motivation to engage in harmful drinking (Biddle, 1986; Kuntsche et al., 2009). However, research by Kuntsche et al., (2011) indicates that the combination of being a mother, employee, and partner is only protective in relation to drinking quantity in countries with high levels of gender equality. This may be because, in more sexist countries, mothers who work may receive less social support and face unsupportive policy environments, contributing to role strain and heightened stress, thus increasing the likelihood of excessive alcohol use as a coping mechanism (Doyal, 1995; Goode, 1960; Kuntsche et al., 2009).

High alcohol consumption amongst women may be particularly harmful as women are more susceptible to several alcohol-related diseases at the same levels of consumption as men (Erol & Karpyak, 2015; White, 2020).. The mixed findings in relation to structural sexism therefore highlight the necessity of understanding how to mitigate alcohol-related harms among women in order to fully leverage the benefits of gender equality. Public health interventions that address modifiable structural factors—like the misplaced use of feminist messages in alcohol advertising (Atkinson et al., 2022)—could help reduce risks and promote healthier behaviors among women. Further, targeted interventions could focus on high-risk groups, such as highly educated women in high-equality states.

Notably, none of the included studies evaluated how gender equality influenced drinking across intersections of SES and race, leaving an important research gap.

#### 4.2.3 Heterosexism

The available evidence suggests there may be a positive association between structural heterosexism and HID for sexual minority men. Structural heterosexism may contribute to HID as a result of minority stress, as an act of resistance or defiance against societal oppression, or because ‘safe spaces’ for LGBTQ+ communities have historically included bars and clubs. Further, discriminatory policy environments may lead to AUD for sexual minorities by restricting sexual minorities’ ability and/or willingness to access to healthcare, mental health services, and substance abuse interventions, perpetuating a cycle of unchecked alcohol use due to inadequate support. These findings are particularly concerning in light of the growing list of legislation limiting the rights of LGBT people in some US states (American Civil Liberties Union, 2024).

As with racism and sexism, the few studies which considered heterogeneity within minoritized groups found differential associations. For example, comprehensive policy protections appear to decrease the probability of HID among sexual minority men, but not sexually minority women (Drabble et al., (2022) while the beneficial effects of civil union legislation on intoxication frequency and HED were seen specifically among women of color and those with lower levels of education, respectively (Everett et al., 2016). The differences may reflect variations in baseline risk, lived experiences of discrimination, differential responsiveness to policy responsiveness, and/or broader social roles. Further research, such as causal mediation analyses for specific groups and intra-categorical qualitative work, may help illuminate the mechanisms driving these differential responses.

#### 4.2.4 Intersectional Discrimination

Most studies considered only one type of discrimination in isolation, for example, racism *or* sexism, which. may provide incomplete or inaccurate picture. Intersectional approaches, which consider how social positions (such as race, gender, and class) and systems of oppression overlap and interact, are more likely to provide a more complete nuanced understanding of inequities (Collins & Bilge, 2020; Combahee River Collective, 1977; Crenshaw, 1989).

A handful of scholars considered the joint impact of multiple forms of structural discrimination simultaneously, using either an intersectional measure (i.e., a single composite exposure combining indicators from different systems of structural oppression) or an intersectional analytic approach (assessing both the individual and joint effects of separate structural discrimination indicators) (Zubizarreta & Beccia, 2025).

Blosnich et al. (2016) constructed an intersectional measure combining policies related to sexual orientation and gender identity discrimination to examine associations with AUD. While they found no significant relationships, a limitation of this approach is that one cannot then disentangle the effects of each structural factor individually, so opposing or interacting effects may be obscured.

In contrast, English (2021) and English et al., (2022) took an intersectional analytic approach, using separate measures of structural racism and LGBTQ-related structural discrimination and assessing both the individual and interactive effects of these exposures. They found that one form of structural discrimination may amplify the effects of another, aligning with prior research on interpersonal discrimination, which has similarly shown that exposure to multiple forms of discrimination can have compounding effects. For example, McCabe et al. (2010) found that U.S. LGB adults who experienced discrimination based on sexual orientation, race, and gender had nearly four times the odds of substance use disorders compared to those reporting no discrimination. This highlights a need for policies and interventions that simultaneously address structural racism and anti-LGBTQ discrimination.

Given the growing but limited evidence on the combined effects of structural discrimination, further structural intersectionality research is critical to advance understanding of how intersecting systems of power and oppression shape alcohol-related inequities. Zubizarreta & Beccia (2025) provide conceptual and methodological considerations for researchers pursuing such research, while Homan et al., (2021) and Bastos et al., (2023) provide concrete examples of structural intersectionality in practice.

#### 4.2.5 Factors contributing to divergent findings

Although some trends have been identified between specific forms of structural discrimination and alcohol-related outcomes, considerable variation across studies remains unexplained. One key contributor to this heterogeneity is the wide range of analytical approaches employed, including differences in study design, statistical modelling, and covariate adjustment, as discussed in Section 3.1.1. Equally important is the diversity in how structural discrimination itself is measured. Even among studies focused specifically on racial segregation, a variety of indicators are used—including Black–White dissimilarity indices, Home Owner’s Loan Corporation (HOLC) scores, trajectories of residential racial segregation across childhood, percentage of Black residents, and instrumental variables. Similarly, although nearly all studies of structural sexism used composite exposures, there is currently no standardized measure, leading to the use of author-constructed measures that vary greatly.

In addition to methodological variability, differences in study populations and social contexts may also contribute to inconsistent results. The effects of structural discrimination are unlikely to be uniform across geographic, temporal, or demographic contexts. Given the relatively small number of studies explicitly examining structural discrimination, we adopted a broad inclusion strategy, not limiting our search by geography, population, or contextual factors. As a result, our review encompasses studies with substantial variation in the underlying structural and social environments—many focused on the U.S. but ranging from national-level analyses to state-or region-specific investigations.

We can illustrate study heterogeneity by comparing two studies with conflicting findings. Egede et al. (2024) and Schwartz et al. (2022) both explore the association between residential segregation and HED, but Egede reports a significant negative association, whereas Schwartz finds no association. These contrasting results may be attributed to several methodological differences. While both use census tract–level indicators of segregation, Egede relies on historical redlining, whereas Schwartz examines childhood segregation trajectories. Their outcome measures also differ: Egede 30-day binge drinking prevalence, without a clear definition of binge drinking, while Schwartz examines annual binge drinking occasions (gender-specific thresholds). The studies also diverge in design— Egede is cross-sectional, while Schwartz is a 27-year cohort study. Finally, their analytic strategies vary: Egede uses tract level structural equation, potentially inflating associations by overlooking individual-level variation; Schwartz uses multilevel modeling with extensive covariate adjustments, with a potential risk of over-adjustment. These differences underscore the need for greater consistency and transparency measuring and modelling of associations between structural discrimination and alcohol.

### 4.3 Limitations

#### 4.3.1 Limitations of this review

As with any systematic review, this study has limitations. One key challenge arose from the complexity and broad scope of our chosen exposure and outcome, which may have led to the exclusion of relevant studies. Our initial database search missed relevant studies that used different terms in their keywords or titles, such as specific exposures (e.g., ‘desegregation’), broader terms that may not be directly related to discrimination (e.g., ‘policy,’ ‘societal factors’), or terms related but not synonymous with discrimination (e.g., ‘structural stigma’). While expanding our keyword list could have captured more studies, the vast number of potential search terms made it inevitable that some relevant studies would be overlooked. To address this limitation, we employed a range of alternative search strategies, including grey literature and reference list screening, which helped identify additional studies not captured by the database search.

The broadness of our exposure and outcome also made cross-study comparisons more challenging, as there was substantial between-study heterogeneity in the exposures and alcohol outcomes considered. This likely also contributed to inconsistencies in study findings. As literature in this field grows, reviews which focus on specific types of structural discrimination, and specific alcohol outcomes, will likely be indicated.

In addition, we included only English language papers, which may have resulted in further exclusion of relevant studies and may help to explain why most studies came from the US. Future reviews of non-English studies are therefore warranted.

Overall, the relative paucity of literature in this field indicates that, although structural discrimination (particularly structural racism) has been identified as a priority by research funders and public health organizations (e.g., American Public Health Association, 2020; Blachman-Demner & Tyus, 2022; Churchwell et al., 2020), further efforts are required to understand the impacts of structural discrimination in relation to alcohol outcomes.

#### 4.3.2 Limitations of the included studies

This review identifies several major gaps in the literature. First, there were gaps in the types of exposures and outcomes studied. Our review identified research on structural sexism, racism, heterosexism, cissexism, or combinations thereof, but no studies addressed other forms of structural discrimination that could influence drinking, such as ableism, ageism, or xenophobia. Further, almost all studies focused on alcohol consumption rather than harm outcomes. This focus on consumption is limiting for several reasons. Social norms and discrimination may influence how individuals report their alcohol use. Self-reported consumption is known to be affected by social desirability bias, which may vary by sociodemographic position (Davis et al., 2010). For example, gender norms may contribute to underreporting among some groups of women. Similarly, individuals experiencing stereotype threat in relation to alcohol use may underreport their drinking to avoid reinforcing negative group-based stereotypes (Green et al., 2007). Focusing on consumption also overlooks the well-documented paradoxical relationship between alcohol consumption and alcohol-related health harms. That is, certain groups, such as those with lower education and some racial and ethnic minoritized groups, face higher alcohol-attributable mortality despite consuming the same, or lower amounts of alcohol (Probst et al., 2021; Zapolski et al., 2014). More studies investigating the associations between structural discrimination and alcohol *harms* could therefore provide deeper insights into health inequities.

Second, there was little consistency in the types of exposures examined across studies. Research on structural racism primarily focused on segregation; studies of sexism often used composite measures emphasizing gender-based inequalities such as income disparities and restrictions on rights; while those on structural heterosexism centered on policy environments. Greater cross-domain learning could expand and refine measurement strategies. For instance, cataloguing state policy environments as indicators of structural sexism presents a key opportunity to expand how sexism is measured (Homan, 2024).

Composite measures may be particularly appropriate for capturing the complex and cumulative impacts of structural discrimination. In contrast, individual measures —such as inequalities in educational attainment—arguably reflect specific forms of *institutional* discrimination (Dean & Thorpe, 2022). Instead of developing new composite measures for each study, alcohol researchers may benefit from agreeing on a standardized set of indicators most relevant to alcohol use and related harms. Utilizing existing resources, like the database of structural racism-related state laws by Agénor et al., (2021), could support this process.

Third, many studies considered outcomes for the minoritized groups only. While centering on oppressed groups is justifiable, considering the impact on dominant, privileged groups can add further value by revealing the breadth of impacts of discrimination. In other words, testing whether there are groups who are unaffected by or even benefit from discrimination and oppression, or whether discrimination is universally (even if differentially) harmful (Homan et al., 2021; Lucas, 2013). This would further help to inform how policies aiming for equality may impact all groups, not just the marginalized.

Fourth, except in the case of structural racism, most studies employed cross-sectional designs, limiting the ability to establish causal relationships between discrimination and alcohol use. It is possible that rather than changes in discrimination leading to changes in drinking, that changes in drinking could lead to changes in discrimination, particularly if the drinking is concentrated within certain social groups. Longitudinal studies that track changes in structural discrimination and alcohol consumption over time would offer stronger evidence that discrimination, rather than other social or cultural factors, drives alcohol-related outcomes. Furthermore, cross-sectional studies can only capture the effects of structural discrimination at a single point in time, whereas the impacts may persist long after the initial exposure. The study of structural discrimination could therefore benefit from developing longitudinal measures that remain consistent over time (Homan, 2019).

Finally, there has been a lack of research examining how different forms of structural discrimination intersect and interact, and how they differentially impact individuals across sociodemographic intersections. A structural intersectionality approach would more accurately reflect the real-world complexities of exposure to structural discrimination, highlighting how multiple forms of disadvantage combine to shape individual experiences.

Novel analytic strategies, that capture both intersectional and life-course effects, offer promising developments in this field (Beccia et al., 2024; Homan et al., 2021; Zubizarreta & Beccia, 2025).

### 4.4 Implications for public health policy and praxis

An understanding of the relationship between structural discrimination and alcohol outcomes is a necessary but insufficient step toward achieving health equity. Future work must be grounded in *critical praxis* — the integration of knowledge and action in the pursuit of social justice (Collins & Bilge, 2020). That is, research should not only seek to understand disparities but also inform meaningful interventions that dismantle systems of structural oppression.

Efforts to reduce structural discrimination are warranted regardless of if they reduce alcohol consumption and related harm, given the broader health consequences of discrimination, and on ethical grounds. Nevertheless, this review identifies some key areas where policies may be targeted if aiming to reduce alcohol consumption and associated harms:

- **Address discriminatory policies and practices:** Positive associations between certain structural discrimination exposures and alcohol outcomes point to potential intervention points. For example, racial inequities in incarceration rates may increase HED among Black Americans. Policies and interventions addressing the underlying drivers of these inequities, such as discriminatory policing practices and bias in the judicial system, may therefore help to reduce alcohol-related harms in these groups.
- **Mitigate the consequences of structural discrimination:** Alongside implementing policy changes to reduce discriminatory practices, efforts should also focus on equipping the most affected areas with resources to mitigate alcohol-related consequences. For example, this may involve ensuring adequate alcohol treatment services in high-incarceration areas and/or offering brief interventions within the criminal justice system (Newbury-Birch et al., 2022).
- **Protect at-risk groups while promoting equity:** Some groups, such as employed women, may experience increased alcohol consumption in contexts of reduced gender discrimination, suggesting a need to support these populations while promoting gender equity.

### 4.5 Future research directions and recommendations

- **Expand research on underexplored forms of structural discrimination:** Future studies should investigate the impacts of forms of structural discrimination that remain largely overlooked, such as ableism, ageism, xenophobia, and other systems of oppression, on alcohol use and related outcomes.
- **Adopt structural intersectionality approaches:** Future studies should examine how multiple systems of discrimination intersect and interact across the life course. To capture the interlocking and cumulative nature of structural—rather than solely institutional—discrimination, researchers should consider using composite index measures that draw on multiple domains, alongside novel analytic approaches (Beccia et al., 2024; Homan et al., 2021; Zubizarreta & Beccia, 2025).
- **Develop consistent cross-domain composite measures of structural discrimination for use in alcohol research:** Researchers should prioritize building or adopting standardized, cross-domain composite measures—such as policy environment indexes—rather than relying on ad hoc exposure metrics (e.g., Homan, 2024; Agénor et al., 2021). These measures should incorporate indicators considered most relevant to alcohol outcomes.
- **Identify and test causal pathways:** Future research should attempt to clarify the key theoretical mechanisms that might underpin the association between specific forms of structural discrimination and specific alcohol-related outcomes. This could begin with a focused review of proposed mechanisms, followed by empirical pathway analyses to test these mechanisms and identify key mediators.
- **Use longitudinal and causal designs:** More longitudinal research is needed to establish temporal and causal relationships between structural discrimination and alcohol outcomes, and to understand the long-term effects of exposure.
- **Investigate alcohol-related harms, not just consumption:** While most existing studies focus on consumption, research should also examine alcohol-related harms— for example, whether structural sexism may contribute to paradoxical patterns in which disadvantaged groups experience greater harms despite similar overall consumption.
- **Consider effects across all population groups:** Future studies should assess the impact of structural discrimination on both marginalized and privileged groups to understand who benefits and who is harmed. This includes exploring how effects differ not only by individual sociodemographic characteristics, but also for specific intersections (e.g., Black women, low-income LGBTQ+ individuals).
- **Examine contextual variations in associations:** Studies are needed that examine how associations vary across different contexts and settings, for example, across urban and rural counties, wet versus dry states, or differing levels of policy enforcement. Future work should also consider local variation within states, as county-or city-level policy contexts may differ substantially from state-level indicators (Lenk et al., 2024).
- **Review of non-English language studies:** Future reviews of non-English studies are warranted to ensure wider coverage of relevant literature.

### 4.6 Conclusion

This review provides a comprehensive overview of current knowledge on how distinct types of structural discrimination impact upon alcohol outcomes, advancing our understanding of this emerging field, and identifying key directions for future research. We found that the relationship between structural discrimination and alcohol-related outcomes is complex and varies based on the type of discrimination, the population affected, and the specific alcohol outcome under consideration. To advance understanding in this field, there must be a consensus on how to operationalize structural discrimination within alcohol studies, along with broader adoption of intersectional and longitudinal approaches.

## Supplementary Material

Supplementary materials

## Figures and Tables

**Table 1:** Search terms by database

**PubMed**	Exposure terms	Outcome terms
	“discriminatory pract*”[tw]	alcohol*[tw]
	“institutional discriminat*”[tw]	drink*[tw]
	“structural discriminat*”[tw]	“Alcohol-Related Disorders”[MeSH Terms]
	“Prejudice”[MeSH Terms]	“Alcohol abstinence”[MeSH Terms]
	“Social Discrimination”[MeSH Terms]	“Alcohol Drinking”[MeSH Terms]
**Embase**	exp social discrimination/	exp alcohol consumption/
	exp social exclusion/	exp alcohol abuse/
	exp prejudice/	exp drinking behavior/
	structural discriminat*.mp.	exp alcoholism/
	institutional discriminat*.mp.	alcohol*.mp.
	discriminatory pract*.mp.	drink*.mp.
**Psycinfo**	exp social discrimination/	exp “alcohol use”/
	exp social exclusion/	exp “alcohol use disorder”/
	exp prejudice/	alcohol*.mp.
	structural discriminat*.mp.	drink*.mp.
	institutional discriminat*.mp.	
	discriminatory pract*.mp.	
**Sociological Abstracts**	MAINSUBJECT.EXACT.EXPLODE (“Discrimination”)	MAINSUBJECT.EXACT.EXPLODE (“drinking behavior”)
	MAINSUBJECT.EXACT.EXPLODE (“Prejudice”)	MAINSUBJECT.EXACT.EXPLODE (“alcohol abuse”)
	noft(“structural discriminat*”)	MAINSUBJECT.EXACT.EXPLODE(“alcoholism”)
	noft(“discriminatory pract*”)	MAINSUBJECT.EXACT.EXPLODE(“intoxication”)
	noft(“institutional discriminat*”)	noft(drink*)
		noft(alcohol*)
**Overton**	discriminat*	Alcohol*
	racism	Drink*
	sexism	
	classism	
	ageism	
	heterosexism	
	prejudice	

**Table 2:** Characteristics of the included studies (n=25).

	n	%
**Discrimination type**		
Racism	11	44
Sexism	7	28
Heterosexism	4	16
Intersectional discrimination	3	12
**Discrimination exposure level** Country	4	16
State	11	44
Below state level (e.g. district, census block) **Outcome(s)**	10	40
Alcohol consumption	17	68
Alcohol-related harm	4	16
Consumption and harm **Region**	4	16
USA	21	84
Multiple countries **Year**	4	16
2006-2010	5	20
2011-2015	4	16
2016-2020	3	12
2021-2024*	13	52
**Study design** Cross-sectional	16	64
Longitudinal	9	36

**Table 3:** Summary of structural racism studies

Racism										
Author	Site	Study	Sample	Design	Discrimination Measure(s)	Outcome	Modelling approach	Adjustments	Key finding(s)	Quality^1^
Chiang et al., 2024	USA, National	National Longitudina l Study of Adolescent to Adult Health (Add Health)	Non-Hispanic Black and White students from a national cohort of adolescents. Youth sample (wave 1): Mean age 15 (SD 1.66), n= 41,269. Youth adult sample (wave 2): Age 28 (SD 1.76), n = 7,845	Longitudina l, 2 waves, 1994-1995 and 2008-2009	**District-level:** school racial segregation in the 1994-95 school year (Black-White dissimilarity Index). Index ranging 0 to 1, with higher values representing higher segregation.	Consumption: Ever drank alcohol in past 12 months (binary); frequency of drinking to the point of drunkenness in past 12 months.	Multilevel regression	**Individual-level:** Youth sample: age, sex, number of other children in grades 7-11 at home (youth), parental education (youth). Young adult sample: age, sex, childhood parental income (young adult), childhood parental marital status (young adult), childhood parental education (young adult). **Environment-level:** Residential segregation, enrolment size, % eligible for free/reduced lunch, school race composition.	**Negative or non-significant associations** *Ever drink*: Higher segregation associated with lower risk of ever drinking for Black respondents in majority non-White schools (youth sample). Non-significant for the White sample and long term outcomes (youth adult sample). *Frequency of drunkenness*: Same findings as for ever drinking.	Fair
Egede et al. 2024	USA, National	Mapping Inequality project	US adults aged 18+ (11,375 census tracts observations)	Cross-sectional	**Census-tract level:** Historic redlining: Home Owner’s Loan Corporation score (1=“best”, 2= “still desirable”, 3=definitely declining”, 4= “hazardous).	Consumption: Prevalence of HED in past 30-days in a census tract	Structural equation model	**Individual-level:** None, analysis at census tract level. **Environment-level:** Population size of census tract in 2010.	**Negative association** *HED:* Historic redlining negatively associated with HED prevalence within a census tract.	Good
Wooda rd et al., 2024	USA, National	Religion and Health in African Americans (RHIAA)	English-speaking Black Americans, aged 21+, Randomly sampled from a nationally representative selection of US census tracts. (n=1,920)	Cross-sectional	**State-level:** Structural racism composite index (residential segregation, gap in incarceration rates, gap in educational attainment, gap in economic indicators, gap in employment status); each individual dimension independently, excluding gaps in employment status.	Consumption: Drinking frequency (days per month among drinkers); HED frequency (events per month among drinkers)	Multilevel regression	**Individual-level:** Age, gender, education, household income, employment status, religious behavior. **Environment-level:** None	**Positive or non-significant associations** *Drinking frequency:* No significant association *HED frequency:* Positively associated with incarceration index. Non-significant for other exposures.	Fair
Schwar tz et al., 2023	USA, National	Add Health	National sample of adolescents, including non-Hispanic Black, non-Hispanic White, and non-Black student of color. Mean age 15.03 (1.67), n= 53,275.	Cross-sectional	**District-level:** school racial segregation in the 1994-95 school year (Black-White dissimilarity Index). Index ranging 0 to 1, with higher values representing higher segregation.	Consumption: Proportion of peers who ever drank; frequency of peer drinking (how often peers drank in past 12 months); frequency of peer HED (how often peers ‘got drunk’ in past 12 months)	Multilevel regression	**Individual-level:** Grade, age, sex, highest parental education, race and ethnicity. **Environment-level:** School racial composition, district urban or rural, geographic region, district total enrolment, district residential segregation.	**Positive, negative, or non-significant associations** *Proportion of peers who ever drank:* Non-significant overall. Positive association for Black students in predominantly White schools. *Frequency of peer drinking:* Non-significant overall. Positive association for Black students in predominantly White schools. *Frequency of peer HED:* Non-significant overall. Negative association for non-Black students of color in predominantly White schools. Non-significant for White students	Good
Kim et al., 2022	USA, National	The Panel Survey of Income Dynamics (PSID)	Black adults (mean age at outcome 28 years, range 18 to 44) who received schooling in districts that were under a desegregation order in 1991. n= 1,053.	Longitudina l, 16 waves (1991-2017)	**District level:** In OLS regression: School racial segregation measured using the Black-White dissimilarity index. In instrument variable (IV) analysis: the proportion of observed schooling years that each child spent in school districts that were released from desegregation.	Consumption: Ever drank; HED status (3+ drinks per day) - both binary.	Single-level regression	**Individual-level:** Sex, household income, parental marital status, birth year, state of residence. **Environment-level:** School district student enrolment, % White, Black and Hispanic students, % eligible for free or reduced lunch, residential segregation.	**Positive or non-significant associations** *Ever drank:* Positive association in IV models, non-significant after adjustment for multiple hypothesis testing. *HED:* Positive association in both OLS and IV models (IV robust to adjustment for multiple testing).	Fair
Schwar tz et al., 2022	USA, National	PSID Transition to Adulthood Supplement	Black Americans (mean birth years between 1991 and 1992). n=1,823	Longitudina l, biennial (followed up to 27 years)	**Census-tract level:** Residential racial segregation trajectories	Consumption: Ever drank (binary); Frequency (binary, drank more than once per week in past year); HED frequency (number of binge-drinking days in past year).	Multilevel regression	**Individual-level:** Average family income across childhood, parental education, parental marital status. **Environment-level:** Average census tract poverty level, population density.	**Positive or non-significant associations** *Ever drank:* Non-significant. *Frequency:* Positive association (higher odds of drinking >1/week for those always living in highly segregated, vs. low segregation, neighborhoods) *HED frequency*: Non-significant	Good
Wang et al., 2022	USA, National	PSID Child Developme nt Supplement (CDS)	Black children aged 5-17 between 1991 and 2014, who received schooling in districts that were under a desegregation order in 1991. (n=511 to 518, outcome dependent)	Longitudina l, 4 waves	**District level:** In OLS regression: school racial segregation measured using the Black-White dissimilarity index. In instrument variable (IV) analysis: the proportion of observed schooling years that each child spent in school districts that were released from desegregation.	Consumption: Ever drank; frequency (drink at least monthly); HED frequency (5+ drinks at least monthly) - all binary measures	Single-level regression	**Individual-level:** Sex, age, family income, parental marital status, birth year, state of residence. **Environment-level:** School district student enrolment, % White, Black and Hispanic students, % eligible for free or reduced lunch, residential segregation.	**Positive or non-significant associations** *Ever drank*: Positive association in IV models. Non-significant in OLS models. *Frequency:* Positive association in IV models. Stratified by sex: positive for Black girls, non-significant for Black boys. Non-significant in OLS models. *HED frequency:* Non-significant in both models.	Good
Dudovi tz et a., 2021	USA, National	Add Health	U.S students in grades 7–12 during the 1994–95 school year, aged 18-32 during survey years. (n = 12,438)	Longitudina l, 3 waves: 1994-1995; 2001-2002; 2008	**School-level:** Proportion of non-Latinx white students, controlling for neighborhood racial composition; Within-school cohorting (Index of Dissimilarity) – higher score indicating greater cohorting.	Harm: Alcohol abuse (2+ episodes of alcohol-related life problems) at wave 1 (age 18-26) and wave 2 (24-32)	Multilevel regression	**Individual-level:** Sex, age, family structure, parental education, household income. **Environment-level:** Neighborhood unemployment, proportion of non-Latinx white residents in census block.	**Positive and negative associations** *Alcohol abuse:* Positive association with % White students, for White students. Non-significant for Black or LatinX. Positive association with within-school racial/ethnic cohorting for White students; negative association for Black and Latinx students.	Fair
Karrike r-Jaffe et al., 2013	USA, National	National Alcohol Survey (NAS)	Random sample of adults with oversampling of sparsely populated states and Blacks and Hispanics. (n = 13,997)	Cross-sectional	**State-level:** Income inequality measured as between-race poverty ratios (Black–White and Hispanic–White)	Consumption and harm: 12-month volume from light drinking; 12-month volume from heavy drinking. Dependence; Alcohol-related consequences (dichotomous variable indicating 2+ problems in past 12 months)	Multilevel regression	**Individual-level:** Gender, age, marital status, educational attainment, employment status, household poverty status, race and ethnicity. **Environment-level:** Neighborhood disadvantage, state median income, geocoding accuracy.	**Positive or moderated associations** *Volume from light drinking:* Positive association with Black-White poverty ratio (White and Black individuals) and Hispanic-White poverty ratio (White and Hispanic individuals) *Volume from heavy drinking:* Positive association with Black-White poverty ratio (White and Black people) *Negative alcohol-related consequences:* Moderated associations - higher racism associated with more negative alcohol related consequences for Black and Hispanic, than White, individuals. *Dependence:* Moderated associations - higher racism associated with more dependence for Black and Hispanic, than White, individuals.	Good
Seffrin et al., 2012	USA (Toleda, Ohio)	Toledo Adolescent Relationshi ps Study	Stratified random sample of Black and White adolescents from 62 schools across 7 school districts. Respondents aged 12-19 at Wave 1. (n= 1,016)	Longitudina l (2 waves)	**Census-block level:** Residential segregation (categorical variable with 3 levels: 0% Black, 50% Black, 75% Black)	Consumption: Changes in drinking frequency (0-“never”, to 8 “more than once a day” over past 12 months)	Multilevel regression	**Individual-level:** Peer socializing, parental supervision, religious importance, disadvantage index, family structure, race, gender, age. **Environment-level:** % black in census block group.	**Positive and negative associations** *Changes in drinking frequency*: Negative association in highly segregated Black communities (less increase in alcohol use in 75% Black vs. 50% Black communities). Positive association in highly segregated White communities (greatest increase in alcohol use in 0% Black communities).	Good
LaVeist et al., 2008	USA (Baltimor e, Maryland & national)	Exploring Health Disparities in Integrated Communiti es (EHDIC) & National Health Interview Survey (NHIS)	Adults from a low-income, urban, racially integrated community & matched participants from a nationally representative sample. (n=2,948)	Cross-sectional	**Neighborhood level:** Residential racial segregation (comparing a ‘racially integrated community’ to a matched general population sample).	Consumption: Current drinking status (drinker vs non-drinker)	Single-level regression	**Individual-level:** Age, gender, smoking status, obesity, education, income, type of insurance. **Environment-level:** None.	No significant difference in odds of being a current drinker between White and African American individuals in racially integrated communities. In NHIS-matched sample (higher discrimination) African Americans had lower odds of being a current drinker than White individuals.	Poor

1 Based on our modified Newcastle-Ottawa Quality Assessment

**Table 4:** Summary of structural sexism studies

Sexism										
Author	Site	Study	Sample	Design	Discrimination Measure(s)	Outcome	Modelling approach	Adjustments	Key finding(s)^2^	Quality^1^
McKetta et al., 2023	USA, National	Monitoring the Future	A subsample of women from a prospective cohort study, taken as a representative cross-section of high-school seniors. Study sample aged 19/20 between 1989 and 2008, followed until 2016. (n = 16,571)	Longitudinal, 9 waves, every 1-2 years	**State-level:** Gender inequality (composite measure including % of male state legislators, and gender ratios for residents living at or above the federal poverty line, adults in the labor force, working adults in management occupations, and self-employed working adults).	Consumption: Drinking frequency (past 30 days); HED over last 2 weeks (binary – none/any)	Multilevel regression	**Individual-level:** Age, marital status, highest educational attainment, religious attendance, rurality, race and ethnicity, father’s education, baseline alcohol use. **Environment-level:** State-level GINI coefficient, poverty rate, rurality, alcohol policy climate, religiosity.	**Negative association** *Drinking frequency:* Negative association (women). *Odds of HED:* Negative association (women). Associations not explored for men. Interactions: Significant interactions between structural sexism and women’s employment status.	Good
McKetta et al., 2022	USA, National	Monitoring the Future	As per McKetta et al. (2023) but also including respondents from initial baseline survey. (N = 20,859). Secondary analysis among men in the same age group.	Longitudinal, 10 waves, every 1-2 years	**State-level:** Gender inequality (composite measure including % of male state legislators, and gender ratios for residents living at or above the federal poverty line, adults in the labor force, working adults in management occupations, and self-employed working adults).	Consumption: Drinking frequency (past 30 days); HED over last 2 weeks (binary – none/any)	Multilevel regression	**Individual-level:** Age, race and ethnicity, paternal education. **Environment-level:** State-level GINI coefficient, poverty rate, population density, alcohol policy climate, religiosity.	**Negative association** *Drinking frequency:* Negative association (men & women; stronger for women). *Odds of HED*: Negative association (men and women; stronger for women). *Mediation effects:* Norms and education (but not depressive symptoms) partially mediated these relationships for women.	Good
Graham et al., 2020	27 countries: Argentina; Australia; Brazil; Canada; Chile; Costa Rica; Czech Republic; Denmark; France; India; Ireland; Italy; Japan; Laos; New Zealand; Nicaragua; Norway; Peru; Spain; Sri Lanka; Sweden; Thailand; Uganda; UK; USA; Uruguay; Vietnam	GENACIS; Gender and Alcohols Harm to Others; European Comparative Alcohol Study	Mix of regional and national sampling frames. Respondents typically aged 18+	Cross-sectional	**Country-level:** Gender Inequality Index, 2017. A composite measure including reproductive health (maternal deaths per 100 000 live births, adolescent birth rate); empowerment (percentage of male and female population aged 25+ with at least some secondary education, percentage of parliamentary seats held by women); and labor market participation (female and male labor force participation rates for persons aged 15+).	Consumption: Drinking status (drinker vs abstainer, past 12 months); Volume (number of standard drinks, past 12 months).	Multilevel regression	**Individual-level:** Age. **Environment-level:** None.	**Negative association** *Drinking status:* Negative association for both men and women (stronger for women). *Volume:* No significant associations. *Interactions:* Significant interaction between gender equality and living with children for men, but not women.	Fair
Roberts, 2012	USA, National	Behavioral Risk Factor Surveillance System, 2005	Telephone survey of adults, representative at the State-level. (n >= 454 drinkers in each sex category/State)	Cross-sectional	**State-level:** Five composite measures: State-level women’s socioeconomic status (SES); Gender equality in socioeconomic status; Reproductive rights; Policies relating to violence against women; Women’s political participation.	Consumption: Drinking status (drinkers vs non-drinkers); Drinking frequency; HED (5+) frequency (continuous variable, taking the natural log of the number of HED days in the past 30) ; Volume (quantity & frequency); Risky drinking (1+ HED episode and volume >60g/30g for men/women). All over past 30 days.	Multilevel regression	**Individual-level:** Age, race, income, education, marital status, employment status. **Environment-level:** Income inequality, median income, % evangelical protestant or Mormon.	**Positive or non-significant associations** *Drinking status:* Positive but not significantly (men & women). *Drinking frequency:* Positive associations for SES-related sexism (men & women). *HED frequency:* Positive associations for sexism related to reproductive rights and violence policy (women). *Volume:* Positive associations for SES-related sexism (men). *Risky drinking:* Positive associations for sexism related to violence policy (women) and reproductive rights (men). I*nteractions:* Significant interactions between gender equality and education level, and between gender equality and employment.	Fair
Kuntsche et al., 2011	16 countries: Australia; Austria; Canada; Czech Republic; Denmark; Finland; France; Germany; Hungary; Netherlands; Norway; Spain; Sweden; Switzerland; UK; USA	GENACIS	Mothers aged 25-49 who consumed alcohol in the past 12 months, sampled from 3 multinational studies. (n=12,454).	Cross-sectional	**Country-level:** Gender-income ratio	Consumption: Quantity (usual amount on a drinking day).	Structural equation model	**Individual-level:** Age, educational attainment. **Environment-level:** Gross National Income (GNI).	**No association** *Quantity:* No association between the gender-income ratio and drinking quantity for ‘housewives’ *Interactions:* Significant interaction between being a partnered working mother and the gender–income ratio.	Fair
Bond et al., 2010	22 countries: Argentina; Australia; Belize; Brazil; Canada; Costa Rica; Denmark; Iceland; India; Isle of Man; Japan; Kazakhstan; New Zealand; Nicaragua; Nigeria; Spain; Sri Lanka; Sweden; Uganda; UK; Uruguay; USA	GENACIS	Men and women, typically aged 18+ (n countries =22)	Cross-sectional	**Country-level:** Six composite measures: Gender Empowerment Measure; Economic Participation & Opportunity; Educational Attainment; Political Participation; Reproductive Autonomy Factor; Violence Against Women Factor.	Consumption: Gender differences in frequency of public drinking; Gender differences in frequency of private drinking.	Multilevel regression	**Individual-level:** Gender, age, marital status. **Environment-level:** Country-level GDP.	**Reduction in gender differences:** *Drinking in public settings:* Lower sexism (higher gender equality in economic participation & opportunity) associated with smaller gender differences in frequency of drinking in public settings. *Drinking in private settings*: Non-significant	Fair
Rahav et al., 2006	29 countries: Argentina; Austria; Brazil; Canada; Costa Rica; Czech Republic; Denmark; Finland; France; Germany; Hungary; Iceland; India; Israel; Italy; Japan; Kazakhstan; Mexico; Netherlands; Nigeria; Norway; Russia; Spain; Sri Lanka; Sweden; Switzerland; Uganda; UK; USA	GENACIS	Men and women, typically aged 18+ (nb. while 29 countries were included in the analysis, the number included in any specific analysis was lower, due to missing values).	Cross-sectional	**Country level:** Gender Empowerment Measure (GEM) Gender Equality Score (GES) Both composite measures.	Consumption and harm: *Gender ratios:* % drinkers; % of drinkers who drink weekly; % of drinkers who drink heavily (>8468 g in past year); % of drinkers who are HED (1+ occasions/ month); liver disease and cirrhosis death rates; alcohol dependency death rates; motor vehicle crashes death rates. *Absolute values (death rates):* Liver disease and cirrhosis; alcohol dependency; motor vehicle crashes; total alcohol deaths.	Correlation	**Individual-level:** None (analysis at country level). **Environment-level:** GDP per capita.	**Positive or no association in relation to gender ratios:** % *drinkers:* positive association* % *weekly drinkers:* non-significant % *heavy drinkers:* non-significant % *HED:* non-significant *liver disease & cirrhosis mortality:* positive association* *alcohol dependency mortality*: positive association* *motor vehicle crash mortality:* positive association* *as sexism increases (GEM/GES decrease), gender differences increase. **Positive associations in relation to absolute death rates:** *Liver disease & cirrhosis*: positive association (men & women) *Alcohol dependency*: positive association (women, GES only). *Total alcohol deaths*: positive association (men & women) NB. Significance at 0.1 level as reported by authors.	Fair

2 To support interpretation, the reported direction of association always relates to structural discrimination and the outcome (rather than, for example, gender equality and the outcome)

**Table 5:** Summary of structural heterosexism studies

Heterosexism										
Author	Site	Study	Sample	Design	Discrimination Measure(s)	Outcome	Modelling approach	Adjustments	Key finding(s)	Quality1
Drabble et al., 2022	USA, National	National A lcohol Surveys (NAS)	Adults aged 18+ from four waves of a cross-sectional probability survey, containing oversamples of racial and ethnic minorities. (n=25,510)	Cross-sectional	**State-level:** A time-varying index of ten state-level policies affecting sexual minorities. Protective policies received a score of +1, negative policies –1. Scores were summed annually per state, ranging from –4 to 6.	Consumption and harm: AUD (DSM-5 AUD 2+); High intensity drinking (binary, 8+ drinks in a day at least once in past year).	Single-level regression	**Individual-level:** Age, partner status, presence of children in the household, race and ethnicity, educational attainment, employment status, survey year. **Environment- level:** None.	**Positive or non-significant associations** *AUD: No* significant associations *HID:* Positive association for sexual minority men. Positive but non-significant associations among heterosexual men and sexual minority women.	Good
Everett et al., 2016	USA, Illinois (Greater Chicago)	Chicago Health and Life Experienc e of Women study (CHLEW)	Black, Latina and White lesbian women (excluding transgender women) aged 18+. Targeted recruitment of underrepresent ed groups. (n=517)	Cross-sectional	**State-level:** Three dichotomous variables indicating if participants were interviewed: i) before Illinois’ civil union bill passed (legalizing civil unions), ii) between passage and enactment, or iii) after enactment.	Consumption and harm: HED (binary, 6+ drinks in a day, past 12 months); Frequency of subjective intoxication (0 ‘never’ to 5 ‘5+ times a week’); Symptoms of potential alcohol dependence (count, 0-5); Experience of 8 adverse drinking consequences past 12 months (count, 0-8)	Single-level regression	**Individual-level:** Sexual identity, age, race and ethnicity, relationship status, presence of child in the household. **Environment-level:** None.	**Positive, negative, and non-significant associations** *HED:* Negative association (increased HED after bill enacted). *Adverse consequences:* Positive association (fewer consequences after both bill passed, and bill enacted). *Frequency of subjective intoxication:* No significant association *Symptoms of dependence:* No significant association *Interactions:* Reduced HED among women with a high school education or less after the bill was signed Fewer experiences of intoxication among Black and Latina women after the bill was signed compared to White women Increased alcohol dependence symptoms among White women after bill passed.	Fair
Hatzenbu ehler et al., 2010	USA, National	NESARC	Nationally representative sample of non-institutionalized adults aged 18+.Sample including both men and women, but analysis not stratified by gender. (n=34,653, including 577 LGB respondents)	Longitudinal, 2 waves	**State-level:** States with constitutional amendments banning gay marriage vs those without	Harm: % change in the prevalence of AUD in past 12 months	Single-level regression	**Individual-level:** Gender, age, race and ethnicity, income, educational attainment, marital status. **Environment-level:** US census region.	**Positive or non-significant associations** *AUD:* Positive association among LGB respondents (increased prevalence of AUDs over time in states with amendments banning gay marriage) Non-significant associations for heterosexuals.	Good
Hatzenbu ehler et al., 2009	USA, National	NESARC	Nationally representative sample of non-institutionalized adults aged 18+. Sample including both men and women but analysis not stratified by gender. (n=34,653, including 577 LGB respondents)	Cross-sectional	**State-level:** 1) Presence of 1+ policies (crime & employment) extending protection to lesbian, gay, and bisexual individuals (dichotomous).	Harm: AUD in the past 12 months	Single-level regression	**Individual-level:** Gender, age, race and ethnicity, income, educational attainment, marital status. **Environment-level:** None.	**Non-significant association** AUD: No significant association.	Good

**Table 6:** Summary of intersectional discrimination studies

Intersectional discrimination										
Author	Site	Study	Sample	Design	Discrimination Measure(s)	Outcome	Modelling approach	Adjustments	Key finding(s)	Quality1
English et al., 2022	USA, natio nal		Purposive internet-based sample of Black and White sexual-minority men aged 16-25. (n=2,033)	Cross-sectional	**State-level:** Racism, heterosexism, and cissexism: Composite State Racism Index (residential segregation, incarceration rates, educational attainment, economic indicators, and employment status, scored 0–100). 2) State Equality Index, (dichotomized, high/low anti-LGBTQ policies)	Consumption: Heavy drinking (3-item AUDIT-C score), continuous measure	Structural equation model	**Individual-level:** Age, sexual identity, relationship status, living situation, contact with living parents or step parents, subjective socioeconomic status, income, employment status, housing instability, insurance coverage, military experience. **Environment-level:** Rural-urban classification.	**Positive or non-significant associations** *Heavy drinking:* Positive association with State Racism index for Black participants. Non-significant for White participants. Non-significant associations with LGBTQ policies for either Black or White participants. *Interactions:* Positive associations between structural racism and heavy drinking were stronger for Black participants living in states with high anti–LGBTQ policies.	Fair
English, 2021	USA, natio nal		Purposive internet-based sample of Black and White sexual-minority men aged 16+ (n=6,916)	Cross-sectional	**State-level:** Racism, heterosexism, and cissexism: Composite State Racism Index (residential segregation, incarceration rates, educational attainment, economic indicators, and employment status, scored 0–100). 2) State Equality Index, (dichotomized, high/low anti-LGBTQ policies)	Consumption: Heavy drinking (3-item AUDIT-C score), continuous measure	Structural equation model	**Individual-level:** Age, sexual identity, relationship status, income, education, employment status, insurance status, housing instability. **Environment-level:** Rural-urban classification.	**Positive or non-significant associations** *Heavy drinking:* Positive association with State Racism index and anti-LGBTQ policies for Black participants. Non-significant for White participants. *Interactions:* Positive associations between structural racism and heavy drinking were stronger for Black participants living in states with high anti–LGBTQ policies.	Fair
Blosnich et al., 2016	USA, natio nal		Purposive sample of transgender veterans (including both those with male and female sex in medical records) from the US Department of Veterans Affairs, with mean age of 54.8, SD 13.2. (n=1,640)	Cross-sectional	**State and municipality level:** Heterosexism and cissexism: Municipal Equality Index (a composite measure evaluating inclusivity of municipal laws, policies, and services for LGBT individuals, scored 0-100, with higher scores representing greater equality); Transgender status in employment non-discrimination laws (yes/no); Transgender status in hate crime laws (yes/no)	Harm: Diagnosed Alcohol Abuse Disorder (AAD)	Single-level regression	**Individual-level:** Age, race and ethnicity, marital status. **Environment-level:** None.	**No significant association** *AAD:* Non-significant	Fair
