# Supplementary material for: The association between macro-level structural discrimination and alcohol outcomes: A systematic review
Source: Soc Sci Med. Author manuscript; Available in PMC 2025 Oct 22. (PMC7618283; doi:10.1016/j.socscimed.2025.118596)
Supplement: Supplementary materials [file EMS209177-supplement-Supplementary_materials.docx]

**Supplementary materials**

**Modified Newcastle-Ottawa Quality Assessment**

| **Cross-sectional studies** | | |
| --- | --- | --- |
| **1. Selection** | | |
| Representativeness of the exposed (high discrimination) cohort | A* | Truly representative of the average person who may be exposed to the discrimination^[[1]](#footnote-1)^ |
|  | B* | Somewhat representative of the average person who may be exposed to the discrimination^[[2]](#footnote-2)^ |
|  | C | Convenience or snow-ball sampling approach in selecting the cohort |
|  | D | No description of the derivation of the cohort |
| Sample size | A* | Justified in a satisfactory manner e.g. with a sample size calculation, or from a large population-based survey |
|  | B | No appropriate justification |
| Non-respondents | A* | Comparability between respondents and non-respondents characteristics is established, and the response rate is satisfactory (>90%) In cases where response is not optional (e.g. secondary data-analysis) comparability refers to differences between individuals dropped due to missing data and those included. |
|  | B | The response rate is unsatisfactory (<90%), or the comparability between respondents and non-respondents is unsatisfactory |
|  | C | No description of the response rate or the characteristics of the responders and non-responders |
| Ascertainment of structural discrimination | A* | A combined multi-dimensional measure (e.g., political participation AND judicial treatment) |
|  | B* | A single dimension measure (e.g. residential segregation alone) well described or multiple measures considered independently |
|  | C | No/poor description |
| **2. Comparability** | | |
| Controls for one or more relevant factor (e.g. age, race) | A* | Yes^[[3]](#footnote-3)^ |
|  | B* | No |
|  | C | Not reported |
| **3. Outcome** | | |
| Assessment of outcome | A* | Secure records (e.g. clinical records, death records) or standardised assessment based on self-report^[[4]](#footnote-4)^ |
|  | B | Not clearly described or known to be not best practice (e.g. a measure of drinking frequency only) |
| Statistical test | A* | Statistical test used to analyse the data clearly described, appropriate and measures of association presented including variance (e.g. CIs) |
|  | B | Statistical test not appropriate, not described, incomplete or unclear. |

*Max score =7*

*Studies are rated from 0–7, with those studies rating 0–3 (poor quality), 4–5 (fair quality), 6–7 (good/high quality).*

| **Cohort studies** | | |
| --- | --- | --- |
| 1. Selection | | |
| Representativeness of the exposed (high discrimination) cohort | A* | Truly representative of the average person who may be exposed to the discrimination1 |
|  | B* | Somewhat representative of the average person who may be exposed to the discrimination2 |
|  | C | Selected group only e.g. convenience and snow ball sampling approach in selecting the cohort |
|  | D | No description of the derivation of the cohort |
| Selection of the non-exposed cohort | A* | Drawn from the same demographic group as the exposed cohort |
|  | B | Drawn from a different source |
|  | C | No description of the derivation of the non exposed cohort |
| Ascertainment of structural discrimination | A* | A multi-dimensional measure (e.g., political participation AND judicial treatment) |
|  | B* | A single dimension measure (e.g. residential segregation alone) well described or multiple measures considered independently |
|  | C | No description / poorly described |
| 1. Comparability | | |
| Study controls for one or more factor of relevance (e.g. age, race) | A* | Yes3 |
|  | B | No |
|  | C | Not reported |
| 1. Outcome | | |
| Assessment of outcome | A* | Secure records (e.g. clinical records, death records) or standardised assessment (e.g. questionnaire of quantity and frequency of alcohol consumption)4 |
|  | B | Not clearly described or known to be not best practice (e.g. a measure of drinking quantity only/ not a standardized measure) |
| Adequacy of follow up of cohorts | A | Subjects lost to follow up unlikely to introduce bias - small number lost - 80% or more follow up or description of those lost suggesting no different from those followed |
|  | B | Follow up rate < 20% or those lost are different from those followed |
|  | C | Not reported |

*Max score = 6*

*Studies are rated from 0–6, with those studies rating 0–2 (poor quality), 3–4 (fair quality), 5–6 (good/high quality).*

Discrepancies in scoring were resolved through discussion until consensus was reached, without the need for third-party adjudication. Most disagreements concerned the representativeness of the study population or the adequacy of the outcome measures. To improve clarity and consistency, additional detail was added to the assessment tool in the form of footnotes. For instance, under ‘assessment of outcome,’ we collaboratively defined what constitutes a ‘standardised assessment based on self-report.’ In a few cases, discrepancies were due to simple errors that were easily resolved upon joint review of the manuscript and did not require further discussion.

1. The demographic group can be a subgroup defined by our demographic strata (sex, age, race and ethnicity) within a state or country; pure low SES samples in specific communities do not meet this criterion. Complete assessment of a birth cohort meets this criterion but not if only a few hospitals were included. Probability samples generally meet this criterion. [↑](#footnote-ref-1)
2. (Probability) sample selected from a geographic level below state level; birth cohort based on selected hospitals only [↑](#footnote-ref-2)
3. If estimates are demographic specific, this criterion is fulfilled [↑](#footnote-ref-3)
4. Standardised measures include:
   1. An outcome of quantity AND frequency (two separate questions combined)
   2. Heavy drinking (a combined quant/ freq. measure with a threshold for heavy drinking)
   3. HED (any reasonable definition)
   4. AUDIT-C (or quant + freq. or binge drinking components) [↑](#footnote-ref-4)
